# Iripin-3, a New Salivary Protein Isolated From *Ixodes ricinus* Ticks, Displays Immunomodulatory and Anti-Hemostatic Properties *In Vitro*

**DOI:** 10.3389/fimmu.2021.626200

**Published:** 2021-03-01

**Authors:** Adéla Chlastáková, Jan Kotál, Zuzana Beránková, Barbora Kaščáková, Larissa Almeida Martins, Helena Langhansová, Tatyana Prudnikova, Monika Ederová, Ivana Kutá Smatanová, Michail Kotsyfakis, Jindřich Chmelař

**Affiliations:** ^1^Department of Medical Biology, Faculty of Science, University of South Bohemia in České Budějovice, České Budějovice, Czechia; ^2^Laboratory of Genomics and Proteomics of Disease Vectors, Institute of Parasitology, Biology Centre of the Czech Academy of Sciences, České Budějovice, Czechia; ^3^Laboratory of Structural Chemistry, Institute of Chemistry, Faculty of Science, University of South Bohemia in České Budějovice, České Budějovice, Czechia

**Keywords:** tick, serpin, X-ray crystallography, blood coagulation, inflammation, adaptive immunity, *Ixodes ricinus*, saliva

## Abstract

Tick saliva is a rich source of pharmacologically and immunologically active molecules. These salivary components are indispensable for successful blood feeding on vertebrate hosts and are believed to facilitate the transmission of tick-borne pathogens. Here we present the functional and structural characterization of Iripin-3, a protein expressed in the salivary glands of the tick *Ixodes ricinus*, a European vector of tick-borne encephalitis and Lyme disease. Belonging to the serpin superfamily of protease inhibitors, Iripin-3 strongly inhibited the proteolytic activity of serine proteases kallikrein and matriptase. In an *in vitro* setup, Iripin-3 was capable of modulating the adaptive immune response as evidenced by reduced survival of mouse splenocytes, impaired proliferation of CD4^+^ T lymphocytes, suppression of the T helper type 1 immune response, and induction of regulatory T cell differentiation. Apart from altering acquired immunity, Iripin-3 also inhibited the extrinsic blood coagulation pathway and reduced the production of pro-inflammatory cytokine interleukin-6 by lipopolysaccharide-stimulated bone marrow-derived macrophages. In addition to its functional characterization, we present the crystal structure of cleaved Iripin-3 at 1.95 Å resolution. Iripin-3 proved to be a pluripotent salivary serpin with immunomodulatory and anti-hemostatic properties that could facilitate tick feeding via the suppression of host anti-tick defenses. Physiological relevance of Iripin-3 activities observed *in vitro* needs to be supported by appropriate *in vivo* experiments.

## Introduction

The European tick *Ixodes ricinus* (Acari: Ixodidae) is an obligate blood-sucking ectoparasite that transmits several medically important pathogens such as Lyme disease spirochetes from the *Borrelia burgdorferi* sensu lato complex and tick-borne encephalitis virus (1). The insertion of the tick hypostome and two chelicerae into host skin disrupts the surrounding tissue and capillaries, to which the host responds by activating a series of physiological defense processes including hemostasis and innate and adaptive immune responses (2–5). Cutaneous tissue injury and tick antigens are sensed by cells in the vicinity of the tick attachment site, such as keratinocytes, fibroblasts endothelial cells, mast cells, macrophages and dendritic cells (3). These cells release pro-inflammatory and chemotactic molecules that stimulate the recruitment of neutrophils and other immune cells to the area of tick feeding (3, 4, 6). Moreover, Langerhans cells and macrophages trap tick antigens and present them to T cells, which triggers T cell proliferation and ultimately results in the development of the acquired immune response (7). If unopposed, the host defense reaction rejects the tick via detrimental effects on tick viability and reproduction (8). Therefore, ticks surpass the host response by secreting hundreds of bioactive molecules via their saliva into the wound (9–11). Since these salivary molecules can target hemostasis and almost every branch of the immune response, they might be useful in the development of novel pharmaceuticals for the treatment of immune-mediated inflammatory diseases, hypercoagulable states, diseases associated with excessive complement activation, or even cancer (11–14). Moreover, tick salivary proteins represent potential targets for the development of anti-tick and/or transmission blocking vaccines (15).

Protease inhibitors form the largest functional group of tick salivary proteins (16). Based on their specificity, tick protease inhibitors can be divided into inhibitors of cysteine proteases (e.g., cystatins) and inhibitors of serine proteases (e.g., Kunitz domain-containing proteins and serpins) (17). Serpins (serine protease inhibitors) are mid-sized proteins consisting of about 330–500 amino acids (18, 19) with a conserved serpin domain and an exposed region near the carboxyl-terminal end referred to as the reactive center loop (RCL) (20). Cleavage of the scissile P1-P1′ bond in the RCL by a target serine protease results in the formation of a covalent serpin-protease complex and permanent inactivation of both the serpin and the protease (18, 20).

Serpins have been identified in many species of hard-bodied ticks of medical and veterinary importance such as *Amblyomma americanum* (21), *Haemaphysalis longicornis* (22), *I. ricinus* (23), *I. scapularis* (24), *Rhipicephalus appendiculatus* (25), and *Rhipicephalus microplus* (26, 27). Some of the functionally characterized tick serpins have been shown to suppress the enzymatic activity of blood clotting factors (mainly thrombin and factor Xa) and consequently inhibit the intrinsic and common coagulation pathways (28–31). Tick serpins that inhibit thrombin and cathepsin G can block platelet aggregation triggered by these two serine proteases (30–33). In addition to anti-hemostatic activities, many of the functionally characterized tick serpins interfere with the host innate immunity, since they inhibit the enzymatic activity of mast cell and neutrophil serine proteases, reduce vascular permeability and paw edema formation, suppress neutrophil migration *in vivo* and attenuate the production of pro-inflammatory cytokines by activated innate immune cells, such as macrophages and dendritic cells (32, 34–37). Last but not least, tick serpins can modify the host adaptive immune response via suppression of T lymphocyte proliferation and inhibition of Th1 and Th17 cell differentiation (35, 37–40). A number of RNA interference and vaccination experiments have demonstrated the important role of tick serpins in successful completion of a blood meal by prolonging the feeding period, reducing engorgement weight, or resulting in higher mortality rates or impaired oviposition (41–45).

To date, only two serpins from the tick *I. ricinus* have been assigned functions: Iris (*I. ricinus* immunosuppressor) (38) and IRS-2 (*I. ricinus* serpin-2) (32). Due to possible confusion arising from the previously used abbreviation IRS for *I. ricinus* serpins (32) (with insulin receptor substrates), we decided to name *I. ricinus* serpins Iripins (*Ixodes ricinus* serpins). Here we present the structural and functional characterization of Iripin-3 (*I. ricinus* serpin-3). Iripin-3 primarily inhibited two trypsin-like serine proteases, kallikrein and matriptase. When tested in various *in vitro* assays, Iripin-3 displayed several distinct functions: it inhibited the extrinsic blood coagulation pathway, attenuated interleukin-6 (IL-6) production by LPS-activated bone marrow-derived macrophages (BMDMs), impaired the survival and proliferation of CD4^+^ T cells, and suppressed the Th1 immune response. The presence of Iripin-3 protein in tick saliva suggests that this serpin could play a role at the tick-host interface by suppressing various aspects of the host defense to *I. ricinus* feeding. Further *in vivo* studies, however, are necessary to confirm herein presented results. Finally, we determined the crystal structure of cleaved Iripin-3 at 1.95 Å resolution.

## Materials and Methods

### Animals

C57BL/6N mice were purchased from Velaz, Ltd (Praha-Lysolaje, Czechia). C3H/HeN mice and OT-II transgenic mice were obtained from Charles River Laboratories (Wilmington, MA). Mice were maintained under standard, pathogen-free conditions in the animal house facility of the Department of Medical Biology, Faculty of Science, University of South Bohemia in České Budějovice, Czech Republic. Guinea pigs utilized for *I. ricinus* feeding and a rabbit used for the production of anti-Iripin-3 antibodies were bred and maintained at the Institute of Parasitology, Biology Centre of the Czech Academy of Sciences (IP BC CAS), Czech Republic. All animal experiments were performed in accordance with the Animal Protection Law of the Czech Republic No. 246/1992 Sb. (ethics approval No. 34/2018) and protocols approved by the Ministry of Education, Youth and Sports of the Czech Republic (protocol No. 19085/2015-3) and the responsible committee of the IP BC CAS. Pathogen-free *I. ricinus* ticks were obtained from the tick colony maintained at the IP BC CAS.

### Bioinformatics Analyses

The molecular weight and isoelectric point of Iripin-3 were computed by ProtParam (46). The presence of a signal peptide was predicted using the SignalP 4.1 server (47). The ScanProsite tool (48) was utilized to identify the serpin signature motif PS00284 as well as two other consensus amino acid motifs N-[AT]-[VIM]-[YLH]-F-[KRT]-[GS] and [DERQ]-[VL]-[NDS]-E-[EVDKQ]-G (26, 49). The reactive central loop together with the amino acid residue at the P1 site were determined based on the eight-residue pattern p17[E]-p16[E/K/R]-p15[G]-p14[T/S]-p13[X]-p12-9[AGS]-p8-1[X]-p1′-4′ [X] (26, 49). NetNGlyc 1.0 (Gupta et al., unpublished) and NetOGlyc 4.0 (50) servers were used to predict potential N-glycosylation and O-glycosylation sites, respectively. To compare Iripin-3 with other known serpins, the Iripin-3 protein sequence was tested against the GenBank database of non-redundant protein sequences using BLASTP (51). Alignment of IRS-2 and Iripin-3 amino acid sequences was conducted with ClustalW (52). Visualization of the alignment and addition of secondary structure elements were performed using ESPript 3.0 (53).

### Crystal Structure Determination

The production of recombinant Iripin-3 in an *Escherichia coli* expression system is detailed in the **Supplementary Materials**. Crystallization experiments were conducted using the sitting-drop vapor diffusion technique, and the obtained crystals were used to collect X-ray diffraction data on the beamline BL14.1 at the BESSY II electron storage ring operated by the Helmholtz-Zentrum Berlin (54). The structure of Iripin-3 was solved by the molecular replacement method, in which the known structure of IRS-2 (Protein Data Bank (PDB) code 3NDA) (32) was used as a search model. The whole procedure of Iripin-3 structure determination, starting with crystallization and ending with structure refinement and validation, is described in detail in the **Supplementary Materials**. Complete data processing and refinement statistics are summarized in **Supplementary Table 1**. Atomic coordinates were deposited in the PDB under accession code 7AHP.

### Phylogenetic Analysis

For the purpose of phylogenetic analysis, the amino acid sequences of 27 tick serpins and one human serpin were retrieved from GenBank. Accession numbers of these sequences are provided in **Supplementary Table 2**. Retrieved sequences were aligned and edited manually using BioEdit 7.2.5 (55). Evolutionary history was deduced from the protein sequences without a signal peptide by using the maximum likelihood method and Jones-Taylor-Thornton (JTT) matrix-based model (56). Initial trees for the heuristic search were obtained automatically by applying the neighbor-joining (57) and BIONJ (58) algorithms to a matrix of pairwise distances estimated using the JTT model, and then the topology with a superior log likelihood value was selected. The reliability of individual branches was determined by bootstrapping. Bootstrap values were calculated for 1000 replicates. Evolutionary analyses were conducted in MEGA X (59).

### Iripin-3 Expression in Ticks

*I. ricinus* nymphs were fed on C3H/HeN mice for 1 day, 2 days, and until full engorgement (3–4 days). *I. ricinus* adult females were fed on guinea pigs for 1, 2, 3, 4, 6, and 8 days. Tick removal from host animals at given time points was followed by the dissection of nymphs and adult female salivary glands, midguts, and ovaries under RNase-free conditions. RNA was isolated from tick tissues using TRI Reagent (Molecular Research Center, Inc., Cincinnati, OH), and 1 μg of total RNA was reverse transcribed into cDNA using the Transcriptor First Strand cDNA Synthesis Kit (Roche Applied Science, Penzberg, Germany) according to the manufacturer's instructions. Five-fold diluted cDNA mixed with FastStart Universal SYBR Green Master (Roche Applied Science) and gene-specific primers were used for the analysis of *iripin-3* expression in the Rotor-Gene 6000 thermal cycler (Corbett Research, Saffron Walden, UK). Cycling conditions were 95°C for 10 min followed by 45 cycles of 95°C for 15 s, 60°C for 10 s and 72°C for 30 s. The relative quantification of *iripin-3* transcripts in tick tissues was performed using the ΔΔCt method (60). The *I. ricinus* gene encoding ribosomal protein S4 (*rps4*, GenBank accession number MN728897.1) was utilized as a reference gene for the calculation of relative expression ratios (61, 62). Nucleotide sequences of forward and reverse primers as well as amplicon lengths are provided in **Supplementary Table 3**.

### Presence of Iripin-3 in Tick Saliva

Polyclonal antibodies against Iripin-3 were produced in a rabbit injected subcutaneously with 100 μg of purified Iripin-3 in 500 μl of complete Freund's adjuvant. The first immunization was followed by another two injections of Iripin-3 in 500 μl of incomplete Freund's adjuvant at 14-day intervals. On day 14 after the last injection, the rabbit was sacrificed, and its blood was collected. Prepared rabbit antiserum to Iripin-3 was subsequently utilized for the detection of Iripin-3 in tick saliva by indirect ELISA and western blotting. The saliva was collected from *I. ricinus* ticks feeding for 6–7 days on guinea pigs as described previously (63). ELISA and western blot analyses are detailed in the **Supplementary Materials**.

### Inhibition of Serine Proteases

Preliminary screening of Iripin-3 inhibitory activity against a set of 17 serine proteases was performed as described previously (32), with the exception of factor VIIa (FVIIa). Human FVIIa (Haematologic Technologies, Inc., Essex Junction, VT) at 20 nM concentration was pre-incubated for 10 min at 30°C with 400 nM Iripin-3 before the addition of 250 μM fluorogenic substrate Boc-QAR-AMC. The assay buffer used consisted of 20 mM Tris, 150 mM NaCl, 0.01% Triton X-100, 5 mM CaCl_2_, and 0.1% polyethylene glycol 6000, pH 8.0. After the determination of the substrate hydrolysis rate, the six most strongly inhibited proteases were chosen for more detailed analysis. The assessment of covalent complex formation between Iripin-3 and selected serine proteases and the determination of second-order rate constants of protease inhibition are detailed in the **Supplementary Materials**.

### Blood Coagulation

The effect of Iripin-3 on blood coagulation was tested by prothrombin time (PT), activated partial thromboplastin time (aPTT), and thrombin time (TT) assays. All chemicals were purchased from Technoclone (Vienna, Austria). Citrated human plasma (Coagulation Control N) was mixed either with 6 μM Iripin-3 or with five different Iripin-3 concentrations and then incubated for 10 min at room temperature. To perform the PT test, 100 μl of plasma with added Iripin-3 was incubated for 1 min at 37°C before the addition of 200 μl of Technoplastin HIS pre-warmed to 37°C. Plasma clotting time was measured on the Ceveron four coagulometer (Technoclone). In the aPTT test, the incubation of 100 μl of plasma mixed with Iripin-3 at 37°C for 1 min was followed by the addition of 100 μl of Dapttin TC. After incubating the mixture of plasma and Dapttin at 37°C for 2 min, 100 μl of 25 mM CaCl_2_ was added to initiate the coagulation cascade. Plasma clotting time was determined as described above. To perform the TT test, 200 μl of plasma mixed with Iripin-3 was incubated at 37°C for 1 min. At the end of incubation, 200 μl of thrombin reagent was added, and plasma clotting time was measured as in the PT and aPTT assays.

### Pro-Inflammatory Cytokine Production by BMDMs

Bone marrow cells were isolated from femurs and tibias of C57BL/6N mice. Both ends of the bones were cut with scissors, and bone marrow was flushed with complete medium. The complete medium was prepared by supplementation of RPMI 1640 medium containing glutamine (Biosera) with 10% heat-inactivated fetal bovine serum (FBS, Biosera), 50 μM 2-mercaptoethanol (Sigma Aldrich, St Louis, MO), 100 U/ml penicillin G (Biosera, Kansas City, MO) and 100 μg/ml streptomycin (Biosera). After erythrocyte lysis in RBC lysis buffer (eBioscience, San Diego, CA), bone marrow cells resuspended in complete medium were seeded into 10 cm Petri dishes and incubated in the presence of 10 ng/ml granulocyte-macrophage colony-stimulating factor (GM-CSF, Sigma Aldrich) at 37°C and 5% CO_2_ for 10 days. On days 4 and 7, non-adherent cells were removed and the medium was replaced with fresh complete medium containing 10 ng/ml GM-CSF. On day 10, adherent cells (macrophages) were collected, resuspended in RPMI 1640 medium supplemented only with 0.5% bovine serum albumin (BSA, Biosera), and seeded into 24-well culture plates (2×10^5^ cells in 500 µl of culture medium per well). After 5 h incubation at 37°C and 5% CO_2_, the medium was replaced with fresh RPMI 1640 medium containing 0.5% BSA, and BMDMs were pre-incubated for 40 min with 3 μM or 6 μM Iripin-3. Finally, 100 ng/ml of LPS (Sigma Aldrich; *E. coli* serotype O111:B4) was added, and macrophages were incubated in the presence of Iripin-3 and LPS for another 24 h. At the end of incubation, cells and cell-free supernatants were collected for RNA isolation and protein quantification, respectively. Relative expression of *Tnf*, *Il6*, and *Il1b* in macrophages was determined by RT-qPCR and concentrations of tumor necrosis factor (TNF), IL-6, and interleukin-1β (IL-1β) cytokines in collected supernatants were measured by DuoSet ELISA Development Kits (R&D Systems, Minneapolis, MN) according to the manufacturer's instructions with only minor modifications. The RT-qPCR analysis is described in detail in the **Supplementary Materials**.

### Splenocyte Isolation and Culture in the Presence of Iripin-3

Spleens harvested from OT-II mice were forced through a Corning 70 μm cell strainer to obtain a single cell suspension. Red blood cells (RBCs) were removed from the suspension by the addition of 1× RBC lysis buffer (eBioscience), and the erythrocyte-free spleen cells were resuspended in RPMI 1640 medium with stable glutamine (Biosera) supplemented with 10% heat-inactivated FBS (Biosera), 50 μM 2-mercaptoethanol (Sigma Aldrich), 100 U/ml penicillin G (Biosera), and 100 μg/ml streptomycin (Biosera). Splenocytes were then seeded into 24-well or 96-well culture plates and pre-incubated with 3 μM or 6 μM Iripin-3 for 2 h. Pre-incubation with Iripin-3 was followed by the addition of ovalbumin (OVA) peptide 323–339 (Sigma Aldrich) at a concentration of 100 ng/ml. Splenocytes were incubated in the presence of Iripin-3 and OVA peptide at 37°C and 5% CO_2_ for either 20 h (assessment of cell survival) or 72 h (analysis of cell proliferation and transcription factor expression).

### Survival of B and T Cells

Mouse splenocytes were seeded into 96-well culture plates (5 x 10^5^ cells in 200 μl of complete medium per well), pre-incubated with Iripin-3, and stimulated with OVA peptide. After 20 h incubation at 37°C and 5% CO_2_, cells were harvested for flow cytometry analysis. First, splenocytes were stained with fixable viability dye eFluor 780 (eBioscience). Subsequently, Fc receptors were blocked with anti-CD16/CD32 antibody (eBioscience, clone 93), and surface antigen staining was performed with following monoclonal antibodies purchased from eBioscience: anti-CD45-PerCP-Cyanine5.5 (clone 30-F11), anti-CD19-PE (clone eBio1D3(1D3)), and anti-CD3e-APC (clone 145-2C11). Finally, the active form of caspase 3 in splenocytes was labeled using the FITC Active Caspase-3 Apoptosis Kit (BD Biosciences). The percentage of live CD19^+^ and CD3e^+^ splenocytes as well as the level of active caspase 3 were analyzed on the BD FACSCanto II flow cytometer using BD FACSDiva software version 6.1.3 (BD Biosciences).

### Proliferation of CD4^+^ T Cells

Erythrocyte-free splenocytes were stained with red fluorescent dye eFluor 670 (eBioscience), which allows monitoring of individual cell divisions. The stained splenocytes were seeded into 96-well culture plates (5 x 10^5^ cells in 200 μl of complete medium per well), pre-incubated with Iripin-3, and stimulated with OVA peptide. Cells were allowed to proliferate for 72 h and then were harvested for flow cytometry analysis. Collected cells were stained with FITC-labelled anti-CD4 monoclonal antibody (clone GK1.5, eBioscience) and propidium iodide (eBioscience), and the percentage of proliferating live CD4^+^ splenocytes was measured on the BD FACSCanto II flow cytometer using BD FACSDiva software version 6.1.3 (BD Biosciences).

### Transcription Factor Expression in CD4^+^ T Cells (RT-qPCR)

Splenocytes were seeded into 24-well culture plates (4.5 x 10^6 ^cells in 500 μl of complete medium per well), pre-incubated with Iripin-3, and stimulated with OVA peptide. At the end of 72 h incubation, non-adherent cells were collected, stained with FITC-labeled anti-CD4 monoclonal antibody (clone GK1.5, eBioscience), and CD4^+^ splenocytes were separated from the rest of the cell population using the S3e Cell Sorter (Bio-Rad Laboratories, Hercules, CA). RNA was extracted from CD4^+^ cells with the help of NucleoSpin RNA isolation kit (Macherey-Nagel, Düren, Germany), and 1 μg of total RNA was reverse transcribed into cDNA using the Transcriptor First Strand cDNA Synthesis Kit (Roche Applied Science). RT-qPCR was performed in the CFX384 Touch thermal cycler (Bio-Rad) by utilizing five-fold diluted cDNA, SsoAdvanced Universal SYBR Green Supermix (Bio-Rad), and gene-specific primers. The PCR cycling conditions were 95°C for 3 min followed by 40 cycles of 95°C for 10 s and 60°C for 30 s. The relative quantification of *Tbx21* (*Tbet*), *Gata3*, *Rorc*, and *Foxp3* transcripts in CD4^+^ splenocytes was performed using Pfaffl's mathematical model (64). Based on the results of geNorm analysis (65), *Actb* and *Gapdh* were utilized as reference genes for the calculation of relative expression ratios. Nucleotide sequences of forward and reverse primers as well as amplicon lengths are given in **Supplementary Table 3**.

### Transcription Factor Expression in CD4^+^ T Cells (Flow Cytometry)

Splenocytes were seeded into 24-well culture plates (2 x 10^6 ^cells in 500 μl of complete medium per well), pre-incubated with Iripin-3, and stimulated with OVA peptide. After 68 h incubation at 37°C and 5% CO_2_, 20 ng/ml of phorbol 12-myristate 13-acetate (PMA; Sigma Aldrich) together with 1 μM ionomycin (Sigma Aldrich) were added to re-stimulate the cells. Brefeldin A (eBioscience) at a concentration of 3 μg/ml was added 1 h later, and splenocytes were incubated in the presence of PMA, ionomycin, and brefeldin A for another 4 h. At the end of incubation, non-adherent cells were collected and stained with fixable viability dyes eFluor 520 and eFluor 780 (eBioscience). Subsequently, Fc receptors were blocked with anti-CD16/CD32 antibody (eBioscience, clone 93), and surface antigen staining was performed with anti-CD4-Alexa Fluor 700 (BD Biosciences, clone RM4-5) and anti-CD25-PerCP-Cyanine5.5 (eBioscience, clone PC61.5) monoclonal antibodies. Surface antigen staining was followed by intracellular staining of transcription factors and cytokine IFN-γ, for which the Foxp3/Transcription Factor Staining Buffer Set (eBioscience) was used in conjunction with following monoclonal antibodies: anti-T-bet-APC (clone eBio4B10 (4B10)), anti-GATA-3-PE (clone TWAJ), anti-RORγt-PE-CF594 (clone Q31-378), anti-Foxp3-PE-Cyanine7 (clone FJK-16s), and anti-IFN-γ-PE (clone XMG1.2). All antibodies were purchased from eBioscience except for the anti-RORγt antibody, which was obtained from BD Biosciences. Analysis was performed on the BD FACSCanto II flow cytometer using BD FACSDiva software version 6.1.3 (BD Biosciences).

### Statistical Analyses

Data are presented in all graphs as mean ± the standard error of the mean (SEM). Differences between the mean values of two groups were analyzed by the unpaired two-tailed *t*-test. Differences between the mean values of three or more groups were analyzed by one-way ANOVA or randomized block ANOVA, which involved two variables: a fixed effect factor (treatment) and a random effect factor/block (an experimental run) (66). In the case of a statistically significant result (p < 0.05), Dunnett's *post hoc* test was performed to compare the mean of a control group with the means of experimental groups. All statistical tests were conducted using the software package STATISTICA 12 (StatSoft, Inc.). Statistically significant differences between groups are marked with asterisks (* p < 0.05, ** p < 0.01, *** p < 0.001, **** p < 0.0001).

## Results

### Iripin-3 Belongs to the Serpin Superfamily

**Figure 1:**
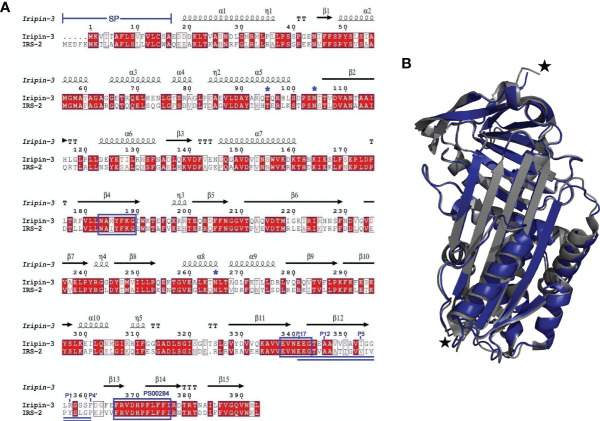
A comparison of the primary, secondary and tertiary structures of Iripin-3 and IRS-2. **(A)** Structure-based sequence alignment of Iripin-3 and IRS-2. Secondary structure elements, which are shown above the aligned sequences, are depicted as spirals (α-helices, 3_10_-helices) and arrows (β-sheets). Both Iripin-3 and IRS-2 possess a signal peptide (SP) at the N terminus of their sequences. Conserved AA motifs PS00284, N-[AT]-[VIM]-[YLH]-F-[KRT]-[GS], and [DERQ]-[VL]-[NDS]-E-[EVDKQ]-G are boxed in blue. The RCLs of both serpins are double underlined. Numbering of amino acid residues in the RCL is based on the standard nomenclature developed by Schechter and Berger (67). Putative N-glycosylation and O-glycosylation sites are marked with blue asterisks. **(B)** Superposition of the cleaved Iripin-3 structure (blue) on the structure of cleaved IRS-2 (gray). Cleavage sites are marked with black stars.

A full-length nucleotide sequence of Iripin-3 was obtained during a salivary gland transcriptome project (16) and was submitted to GenBank under accession number GADI01004776.1. This sequence, consisting of 1182 base pairs, encodes a 377-amino acid (AA) protein with predicted molecular weight of approximately 42 kDa and with theoretical isoelectric point (pI) 5.23. The SignalP 4.1 server found a 16-AA signal peptide at the N terminus of the protein sequence (**Figure 1A**), which indicates that Iripin-3 is a potentially secreted protein. Using ScanProsite, the serpin signature motif PS00284 was identified at AA positions 366-376 (**Figure 1A**). Moreover, two other serpin consensus AA motifs N-[AT]-[VIM]-[YLH]-F-[KRT]-[GS] and [DERQ]-[VL]-[NDS]-E-[EVDKQ]-G were recognized: NAMYFKG at AA positions 183-189 and EVNEEG at AA positions 338-343 (**Figure 1A**), suggesting that Iripin-3 belongs to the serpin superfamily. The hinge region of the Iripin-3 RCL has glycine at the P15 position, threonine at the P14 position, and residues with short side chains (alanine and valine) at positions P12-P9 (**Figure 1A**), which correspond to the RCLs of inhibitory serpins (68). The P1 site is occupied with the basic amino acid residue arginine (**Figure 1A**), suggesting Iripin-3 might target trypsin-like rather than chymotrypsin-like or elastase-like serine proteases (69). Using NetNGlyc 1.0 and NetOGlyc 4.0 servers, the Iripin-3 AA sequence was predicted to contain two potential N-glycosylation sites (N-X-[S/T]) and one putative O-glycosylation site (**Figure 1A**).

### Iripin-3 Adopts a Typical Serpin Fold

**Figure 2:**
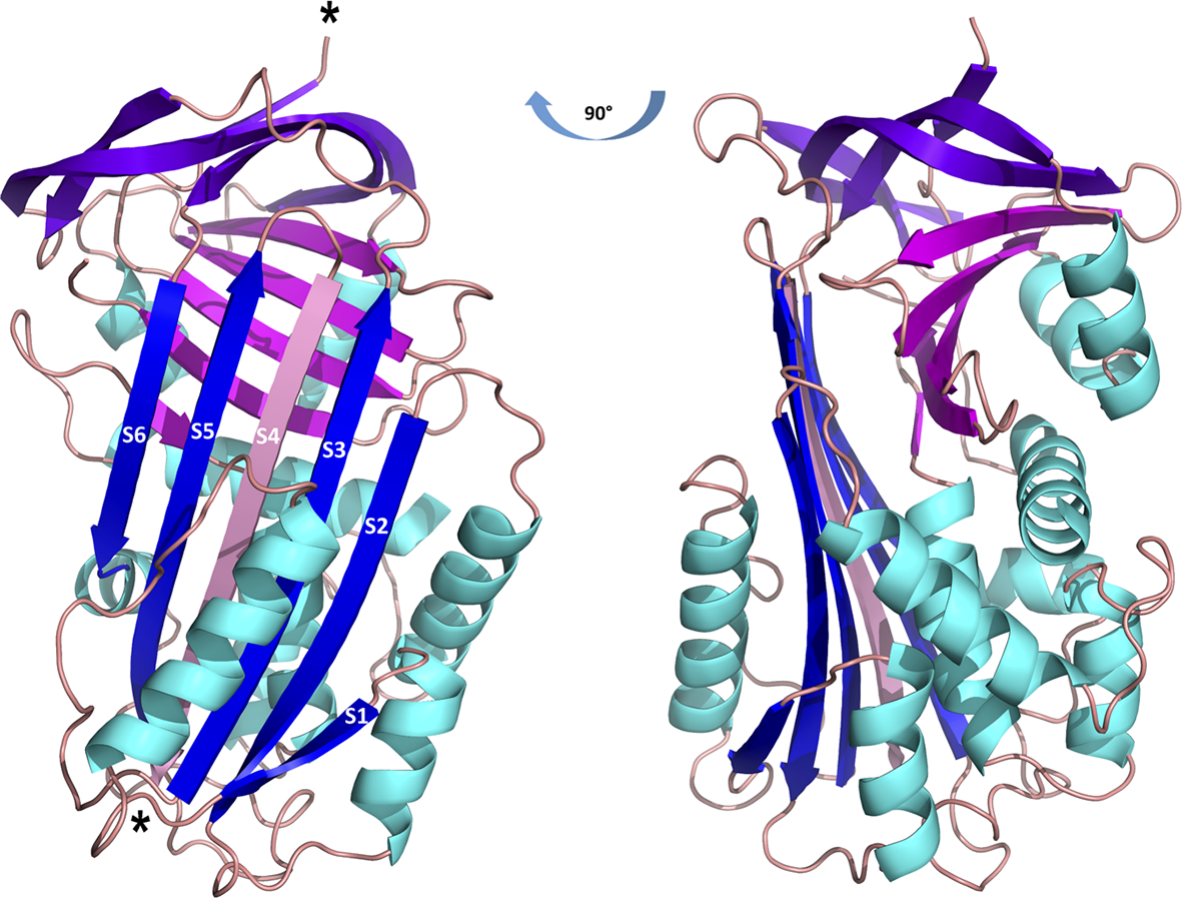
Cartoon representation of the structure of cleaved Iripin-3. α-helices are colored cyan, β-sheet A is blue, β-sheet B is magenta, β-sheet C is purple, and loops are colored wheat. The insertion of the RCL hinge region between β-strands S3 and S5 (depicted in blue) resulted in the formation of an additional β-strand S4 (depicted in pink). Cleavage sites are marked with asterisks.

Employing X-ray crystallography, we determined the 3D structure of Iripin-3 at 1.95 Å resolution. The crystal used exhibited symmetry of the *P*6_2_22 space group and contained one molecule in the asymmetric unit with a solvent content of 42.68%. The tertiary structure of Iripin-3 matched the 3D structures of other serpins, including the tick serpin IRS-2 (**Figure 1B**), with which it had the highest sequence similarity of all the serpin structures currently deposited in the PDB. More specifically, the Iripin-3 tertiary structure was composed of ten α-helices and three β-sheets, which were sequentially arranged in the order α1-β1-α2-α3-α4-α5-β2-α6-β3-α7-β4-β5-β6-β7-β8-α8-α9-β9-β10-α10-β11-β12-β13-β14-β15 (**Figures 1A**, **2**). The sheet A consisted of six β-strands (β2, β3, β4, β10, β11, β12), sheet B of five β-strands (β1, β7, β8, β14, β15), and sheet C of four β-strands (β5, β6, β9, β13) (**Figure 2**). Iripin-3 in the crystal adopted a conformation known as the relaxed (R) state, since its RCL was probably cleaved by some contaminating proteases before or during the crystallization experiment. A protein sample can contain traces of contaminating cysteine and serine proteases, as demonstrated previously (70). The cleavage of the RCL led to the insertion of the RCL hinge region into the β-sheet A as an additional β-strand S4 (**Figure 2**). The 3D structure of Iripin-3 contained 367 amino acid residues. The first 19 residues, which basically corresponded to the signal peptide of the protein, were missing. Moreover, the region _356_LRSGSFD_362_, in which the cleavage occurred, could not be modelled in the Iripin-3 structure due to its absence in the electron-density map. To compare the tertiary structure of Iripin-3 with that of IRS-2, the molecular structure of Iripin-3 was superposed with Cα atoms of IRS-2 with root-mean-square deviation of 0.8085 Å. The secondary structure elements were well conserved in both serpins, but there was a certain degree of divergence in disordered loop regions (**Figure 1B**).

### Iripin-3 Is Most Closely Related to Serpins From *I. scapularis*

**Figure 3:**
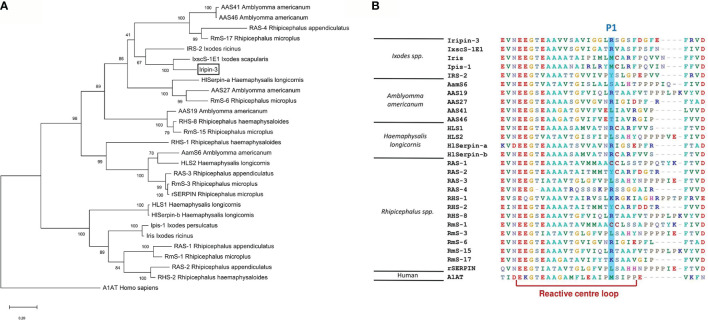
Phylogenetic analysis of selected tick serpins. Protein sequences of previously characterized tick serpins were aligned and analyzed to determine phylogenetic relationships. **(A)** A phylogenetic tree was built using the maximum likelihood method and JTT matrix-based model. Alpha-1-antitrypsin (A1AT) was utilized as an outgroup to root the tree. The branch length represents the number of substitutions per site. The reliability of individual branches, assessed by bootstrapping, is expressed as a percentage of trees in which a given topology was present out of 1,000 replications. Iripin-3 is boxed. **(B)** Alignment of reactive center loop (RCL) regions of 27 tick serpins and one human serpin was performed using BioEdit. RCLs were determined based on the eight-residue pattern p17[E]-p16[E/K/R]-p15[G]-p14[T/S]-p13[X]-p12-9[AGS]-p8-1[X]-p1′-4′ [X] typical for inhibitory serpins (68). Amino acid residues at the predicted P1 site are highlighted in blue.

The BLASTP search of the GenBank non-redundant protein sequences identified three *I. scapularis* serpins (accession numbers XP_029826754.1, EEC19555.1, and AAV80788.1) whose sequences were highly similar to the Iripin-3 sequence (percentage identities 95.4%, 94.9%, and 93.6%, respectively). These homologs have not been functionally characterized. The phylogenetic relationship of Iripin-3 with 26 tick serpins, whose function was deciphered either by using recombinant protein or at least by gene knockdown via RNA interference in ticks, was determined by using the maximum likelihood method and JTT matrix-based model. The resulting phylogenetic tree, with human alpha-1-antitrypsin as an outgroup, showed two distinct groups of tick serpins (**Figure 3A**). The first group at the bottom of the tree included eight serpins without a signal peptide with presumably intracellular function (**Figure 3A**). Notably, these serpins usually contained one or more cysteines and methionines in their RCL (**Figure 3B**). The second, larger group at the top of the tree comprised 19 serpins with a signal peptide, including Iripin-3 (**Figure 3A**). Iripin-3 formed a small branch with one serpin from *I. scapularis* (IxscS-1E1) and one serpin from *I. ricinus* (IRS-2) (**Figure 3A**). In addition to the construction of the phylogenetic tree, we aligned the RCLs of the serpins used in the phylogenetic analysis (**Figure 3B**). Serpins that clustered together usually had similar RCLs, and the RCL of Iripin-3 resembled that of IxscS-1E1 (**Figure 3B**).

### Iripin-3 Is Expressed in Feeding Ticks and Is Secreted Into Tick Saliva

**Figure 4:**
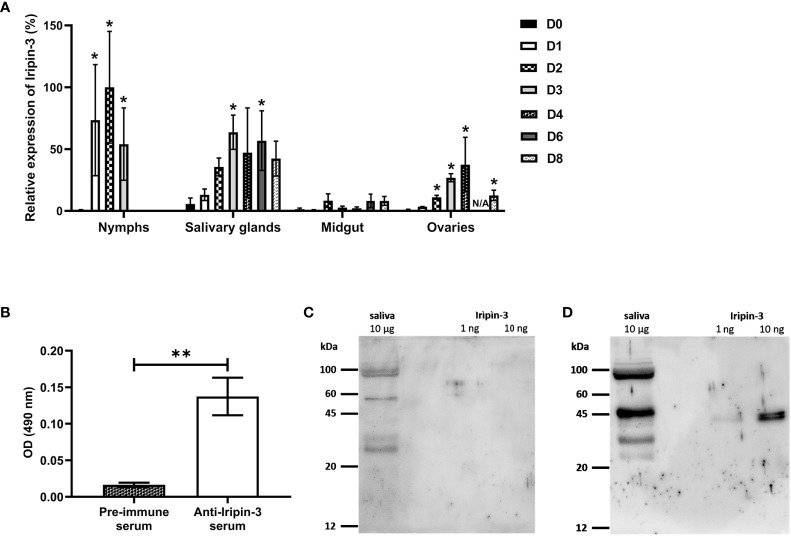
*Iripin-3* transcription in *I*. *ricinus* ticks is increased in response to blood feeding, and Iripin-3 protein is present in the saliva of feeding ticks. **(A)** *Iripin-3* mRNA expression in nymphs and in the salivary glands, midguts and ovaries of adult females feeding for 1 (D1), 2 (D2), 3 (D3), 4 (D4), 6 (D6), and 8 (D8) days or not feeding at all (D0). In nymphs, the last column represents fully engorged ticks that completed their blood meal in 3 or 4 days. N/A – data not available. Relative expression values were calculated using the ΔΔCt (Livak) method (60), with *rps4* serving as a reference gene. A group with the highest *iripin-3* expression (nymphs feeding for 2 days) was utilized as a calibrator during calculations, and its expression value was set to 100%. Data are presented as mean of three biological replicates ± SEM. Statistically significant induction (p < 0.05) of *iripin-3* expression as compared to unfed ticks is marked with an asterisk. **(B)** ELISA results expressed as optical density (OD) values measured after exposure of tick saliva to either rabbit pre-immune serum or rabbit antiserum to Iripin-3. Data are presented as mean ± SEM of three values (**p < 0.01). **(C, D)** Tick saliva (10 μg) and Iripin-3 (1 ng or 10 ng) were resolved by SDS-PAGE and transferred to PVDF membranes. The membranes were incubated with rabbit pre-immune serum **(C)** or rabbit antiserum to Iripin-3 **(D)**.

In order to see how *iripin-3* expression changes during blood feeding, nymphal and adult ticks were allowed to feed on blood from host animals for various periods of time, and the amount of *iripin-3* transcript in tick tissues was subsequently determined by RT-qPCR. Overall, *iripin-3* expression was significantly induced in response to blood feeding in nymphs as well as in the salivary glands and ovaries of adult females (**Figure 4A**). In adults, the highest levels of *iripin-3* mRNA were detected in the salivary glands (**Figure 4A**). To prove the presence of Iripin-3 protein in tick saliva, we collected saliva from ticks that were feeding for 6 to 7 days on guinea pigs. By ELISAs, markedly higher optical density values were obtained after exposure of tick saliva to anti-Iripin-3 serum than to pre-immune serum (**Figure 4B**), suggesting that Iripin-3 is a salivary protein. This result was further confirmed by western blotting. Rabbit pre-immune serum did not recognize recombinant Iripin-3, and there was no band of appropriate size (around 42 kDa) in tick saliva (**Figure 4C**). Conversely, the use of anti-Iripin-3 serum led to the recognition of recombinant Iripin-3 and appearance of an approximately 45 kDa band in tick saliva, which might represent native Iripin-3 (**Figure 4D**). The difference in the sizes of native and recombinant Iripin-3 was probably caused by the fact that native Iripin-3 is glycosylated, whereas recombinant Iripin-3 was prepared in the *E. coli* expression system and therefore lacks glycosylation. The other bands with sizes greater or less than 45 kDa that appeared in the lanes with tick saliva after exposure of membranes to either pre-immune serum or anti-Iripin-3 serum are most likely a result of non-specific binding of antibodies to some components of tick saliva (**Figures 4C, D**).

### Iripin-3 Primarily Inhibits Kallikrein and Matriptase

**Figure 5:**
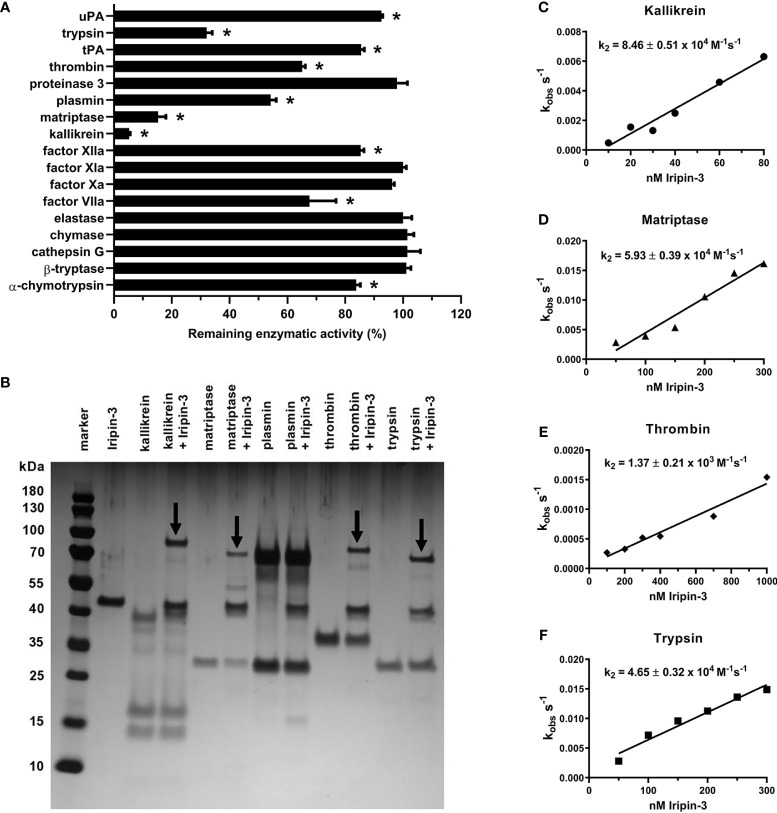
Iripin-3 suppresses the enzymatic activities of kallikrein, matriptase, thrombin, and trypsin through the classic serpin inhibitory mechanism. **(A)** The residual enzymatic activities of 17 selected serine proteases in the presence of 400 nM Iripin-3. The experiment was performed in triplicate, and data are expressed as mean ± SEM. The enzymatic activities of individual proteases in the absence of Iripin-3 (control groups) were considered as 100%, and differences between control groups and Iripin-3-treated groups were analyzed by the unpaired two-tailed *t*-test. Enzymes labelled with an asterisk were inhibited with statistical significance (p < 0.05). **(B)** Formation of SDS- and heat-stable complexes between Iripin-3 and kallikrein, matriptase, plasmin, thrombin, and trypsin. Proteins were resolved on 4 to 12% NuPAGE Bis-Tris gels and visualized by silver staining. Covalent complexes between Iripin-3 and target proteases are marked with black arrows. **(C–F)** The apparent first-order rate constant *k_obs_* was plotted against Iripin-3 concentration, and linear regression was performed to obtain the line of best fit. The slope of the line represents the second-order rate constant *k_2_* for the inhibition of kallikrein **(C)**, matriptase **(D)**, thrombin **(E)**, and trypsin **(F)** by Iripin-3. For each determination, the standard error of the slope is given.

An initial screen for Iripin-3 inhibitory activity was carried out against 17 different serine proteases. Statistically significant reductions in enzymatic activity were observed for ten proteases, but only six of these, namely kallikrein, matriptase, trypsin, plasmin, thrombin, and FVIIa, had their proteolytic activity reduced by >20% (**Figure 5A**). Iripin-3 formed covalent complexes, typical for the serpin “suicide” mechanism of inhibition (71), with kallikrein, matriptase, thrombin, and trypsin, as shown by SDS-PAGE (**Figure 5B**). There was no visible complex between Iripin-3 and plasmin on the gel (**Figure 5B**). It is possible that the complex was hidden within an approximately 70 kDa protein band, which was also present in the lane with plasmin only (**Figure 5B**). Moreover, no SDS- and heat-stable complex was formed between Iripin-3 and FVIIa in the absence or presence of tissue factor under given conditions (**Supplementary Figure 1**), suggesting Iripin-3 probably does not reduce the proteolytic activity of FVIIa through the classic serpin inhibitory mechanism. Finally, the second-order rate constants *k_2_* for the interactions between Iripin-3 and kallikrein, matriptase, thrombin, and trypsin were measured by a discontinuous method under pseudo first-order conditions. Iripin-3 most potently inhibited kallikrein with *k_2_* = 8.46 ± 0.51 x 10^4^ M^-1^ s^-1^ (**Figure 5C**). The *k_2_* for the interactions between Iripin-3 and matriptase and trypsin were determined as 5.93 ± 0.39 x 10^4^ M^-1^ s^-1^ and 4.65 ± 0.32 x 10^4^ M^-1^ s^-1^, respectively (**Figures 5D, F**). Thrombin was inhibited by Iripin-3 with the lowest potency (*k_2_* = 1.37 ± 0.21 x 10^3^ M^-1^ s^-1^) (**Figure 5E**). Interface analysis between the active sites of matriptase, thrombin, kallikrein and trypsin and the P4-P4′ part of Iripin-3 RCL revealed possible polar interactions that could indicate the binding selectivity of Iripin-3 for target proteases (**Supplementary Figure 2**). The strongest interaction with the catalytic triad was calculated for matriptase, followed by trypsin, kallikrein and thrombin (data not shown). According to this analysis, thrombin and kallikrein should be inhibited by Iripin-3 with similar potency. This, however, was not supported by enzyme-substrate kinetic analyses (**Figures 5C–F**), in which kallikrein displayed 60 times higher *k_2_* value than thrombin. Therefore, the specificity of Iripin-3 is probably dependent on more factors. As shown in **Supplementary Figure 3**, matriptase and trypsin have open and shallow active sites, easily accessible to various substrates, including Iripin-3 RCL. Thrombin and kallikrein, on the other hand, possess narrower and deeper cavities with the catalytic triad (**Supplementary Figure 3**). It is possible that some subtle differences in spatial arrangement hinder the access of Iripin-3 RCL to the thrombin's active site, while facilitating its access to the kallikrein's active site cleft.

### Iripin-3 Prolongs Plasma Clotting Time in the Prothrombin Time Assay

**Figure 6:**
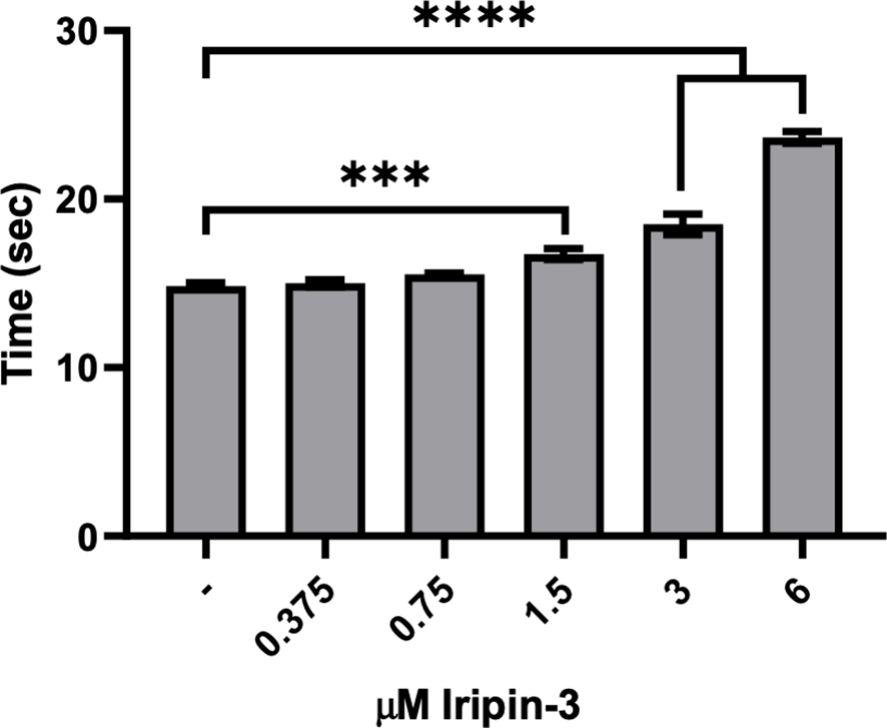
Iripin-3 inhibits the extrinsic pathway of blood coagulation. Human plasma was treated with no Iripin-3 or with 0.375, 0.75, 1.5, 3, and 6 μM Iripin-3 and the time required for blood clot formation in the prothrombin time assay was subsequently determined on a coagulometer. Data are presented as mean ± SEM of three independent experiments (***p < 0.001, ****p < 0.0001).

Since tick serpins commonly inhibit the host coagulation system (72), we tested the effect of Iripin-3 on the extrinsic coagulation pathway, intrinsic coagulation pathway, and common coagulation pathway by using prothrombin time (PT), activated partial thromboplastin time (aPTT), and thrombin time (TT) tests, respectively (73). Iripin-3 at 6 μM final concentration did not significantly prolong plasma clotting time in the aPTT and TT assays (data not shown). However, there was a statistically significant delay in blood clot formation in the PT test when plasma was treated with 1.5, 3, and 6 μM Iripin-3 (**Figure 6**). The highest Iripin-3 concentration prolonged the prothrombin time by 8.8 s when compared to control plasma (**Figure 6**). These results therefore indicate that Iripin-3 slightly inhibits the extrinsic pathway while not affecting the intrinsic and common pathways of blood coagulation.

### Iripin-3 Decreases Production of IL-6 by BMDMs

**Figure 7:**
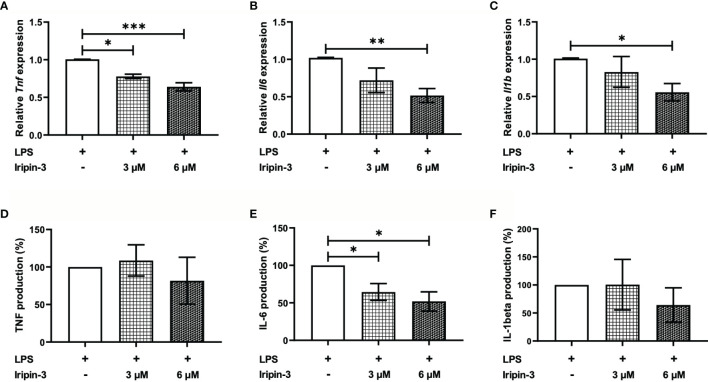
Iripin-3 inhibits the expression of pro-inflammatory cytokines in LPS-stimulated BMDMs. Macrophages derived from bone marrow cells isolated from C57BL/6N mice were pre-incubated with 3 μM or 6 μM Iripin-3 for 40 min and were then stimulated with LPS (100 ng/ml) for 24 h. **(A–C)** At the end of 24 h incubation, cells were harvested for RNA extraction and the expression of *Tnf* **(A)**, *Il6* **(B)**, and *Il1b* **(C)** was determined by RT-qPCR. Relative expression values were calculated using the delta-delta Ct (Livak) method (60), with *Gapdh* serving as a reference gene. Cells incubated only in the presence of LPS were utilized as a calibrator during calculations. Data are presented as mean ± SEM of four independent experiments (*p < 0.05, **p < 0.01, ***p < 0.001). **(D–F)** Supernatants were collected, and TNF, IL-6, and IL-1β concentrations in these supernatants were measured by sandwich ELISA. TNF **(D)**, IL-6 **(E)**, and IL-1β **(F)** production by Iripin-3-treated BMDMs is expressed as the percentage of the cytokine production by control macrophages, since there were large differences in the concentrations of the same cytokine between three independent repeats of the experiment. Data are expressed as mean ± SEM, and statistically significant differences (p < 0.05) are marked with an asterisk.

Serpins secreted in tick saliva can facilitate blood meal uptake not only by inhibiting coagulation but also by suppressing host inflammatory responses (37, 72, 74). Therefore, we next investigated whether Iripin-3 attenuates pro-inflammatory cytokine production by LPS-stimulated BMDMs. The production of TNF, IL-6, and IL-1β was assessed at the mRNA level by RT-qPCR as well as at the protein level by ELISA. Iripin-3 caused a dose-dependent and statistically significant reduction in the transcription of all three genes (**Figures 7A–C**). However, decreases in the transcription of *Tnf* and *Il1b* did not result in corresponding changes in the concentrations of these two pro-inflammatory cytokines at the protein level (**Figures 7D, F**). Conversely, Iripin-3 was an efficient inhibitor of both IL-6 synthesis and secretion (**Figure 7E**).

### Iripin-3 Impairs B and T Cell Viability *In Vitro*

**Figure 8:**
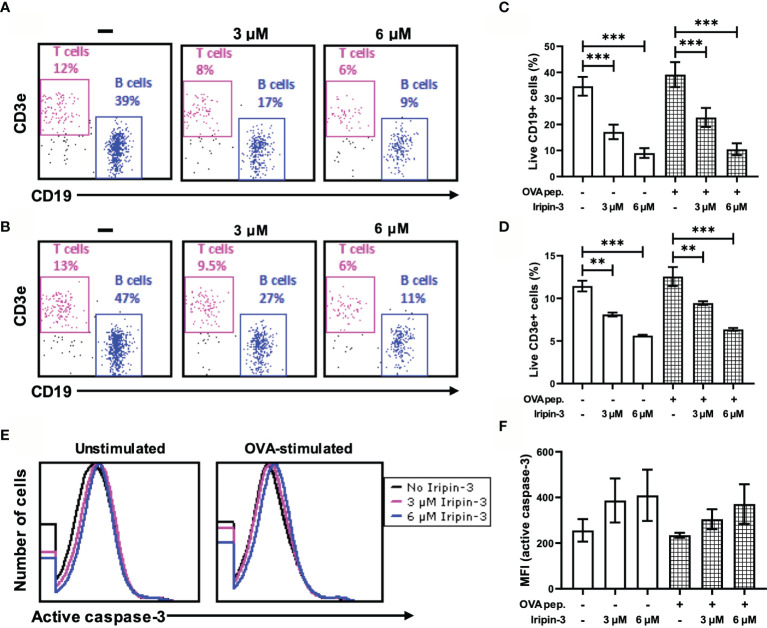
Iripin-3 reduces B and T cell viability and does not significantly alter active caspase-3 levels. **(A, B)** Dot plots depicting the percentage of live CD45^+^CD19^+^ cells (B cells) and live CD45^+^CD3e^+^ cells (T cells) in unstimulated splenocytes **(A)** or OVA peptide-stimulated splenocytes **(B)**. Splenocytes were not treated with Iripin-3 (left) or were treated with 3 μM (middle) or 6 μM (right) Iripin-3. **(C, D, F)** The percentage of live B cells **(C)**, live T cells **(D)**, and median fluorescence intensity (MFI) corresponding to the level of active caspase-3 **(F)** after incubating the splenocytes for 20 h in the absence of Iripin-3 or in the presence of 3 μM and 6 μM Iripin-3. The cells were left either unstimulated or were stimulated with 100 ng/ml of OVA peptide. Data are presented as mean ± SEM of three independent experiments (**p < 0.01, ***p < 0.001). **(E)** Histograms showing the level of active caspase-3 in either unstimulated splenocytes (left) or splenocytes stimulated with OVA peptide (right). Splenocytes were incubated for 20 h without Iripin-3 or were treated with 3 μM or 6 μM Iripin-3.

In addition to inhibiting innate immune mechanisms, tick serpins can alter the host adaptive immune response (35, 37, 72). First, we tested whether Iripin-3 had an effect on B and T lymphocyte viability. Incubation of splenocytes derived from OT-II mice for 20 h in the presence of two different concentrations of Iripin-3 (3 μM and 6 μM) resulted in a pronounced dose-dependent reduction in the viability of both B cells (CD45^+^ CD19^+^ splenocytes) and T cells (CD45^+^ CD3e^+^ splenocytes), with B cell survival more negatively affected by the serpin presence than T cell survival (**Figures 8A–D**). B and T cell viability was impaired irrespective of whether the splenocytes were left unstimulated or were stimulated with OVA peptide (**Figures 8C, D**). Conversely, Iripin-3 did not reduce the viability of BMDMs or dendritic cells (**Supplementary Figures 4A, B**), and the viability of LPS-activated neutrophils was impaired only in the presence of the highest (6 μM) concentration of Iripin-3 (**Supplementary Figure 4C**). Therefore, Iripin-3 might selectively induce B and T cell death. To investigate the possibility that Iripin-3 triggers lymphocyte apoptosis, we measured active caspase-3 levels in both unstimulated and OVA peptide-stimulated splenocytes. Treatment of splenocytes with Iripin-3 did not lead to a statistically significant increase in the level of active caspase-3 (**Figures 8E, F**). Therefore, Iripin-3 probably does not induce B and T cell death through activation of a caspase-3-dependent pathway.

### Iripin-3 Inhibits *In Vitro* CD4^+^ T Cell Proliferation

**Figure 9:**
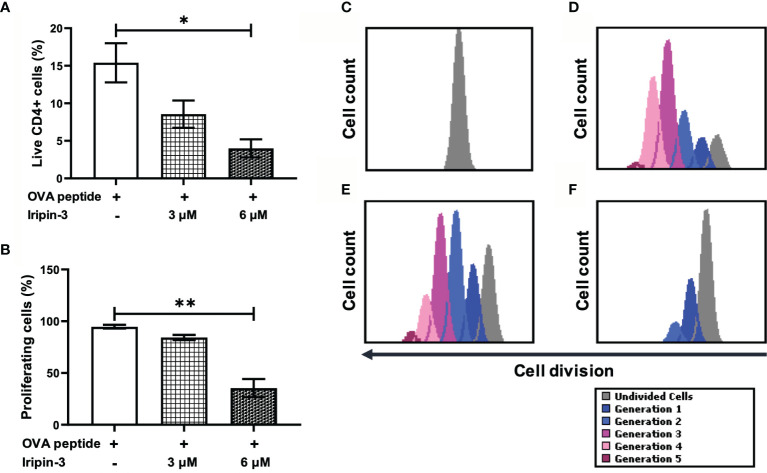
Iripin-3 impairs the survival and proliferation of CD4^+^ splenocytes. **(A, B)** The percentage of live CD4^+^ cells **(A)** and the percentage of proliferating live CD4^+^ cells **(B)** after exposure to 3 μM or 6 μM Iripin-3. Cells not treated with Iripin-3 were used as control. After 2 h pre-incubation with Iripin-3, cells were cultured in the presence of OVA peptide (100 ng/ml) for 72 h. Data are presented as mean ± SEM of three independent experiments (*p < 0.05, **p < 0.01). **(C–F)** Histograms showing the number of live CD4^+^ cells that managed to divide once (blue), twice (light blue), 3 times (pink), 4 times (rose), 5 times (plum), or did not divide at all (gray) within the 72 h culture period. Cells were incubated in the absence of Iripin-3 and OVA peptide **(C)**, in the presence of OVA peptide only **(D)**, or were treated with the combination of 3 μM Iripin-3 and OVA peptide **(E)** or 6 μM Iripin-3 and OVA peptide **(F)**.

Since Iripin-3 reduced T cell viability, we tested whether it also affected the survival and proliferation of CD4^+^ helper T cells. OT-II splenocytes were pre-incubated with 3 μM or 6 μM Iripin-3 for 2 h before being stimulated with OVA peptide for 72 h. Propidium iodide staining in combination with the application of anti-CD4 antibody revealed a lower percentage of live CD4^+^ cells in Iripin-3-treated groups than in the control group (**Figure 9A**), suggesting Iripin-3 has a negative effect on CD4^+^ T cell viability. After the exclusion of dead cells, we assessed the proliferation of CD4^+^ T cells. Unstimulated CD4^+^ cells did not proliferate at all (**Figure 9C**), whereas addition of OVA peptide triggered proliferation in approximately 95% of cells (**Figures 9B, D**). Treatment with Iripin-3 caused a dose-dependent decrease in CD4^+^ splenocyte proliferation (**Figure 9B**). While about 84% of cells proliferated in the presence of 3 μM Iripin-3 (**Figures 9B, E**), only 35% of cells were capable of proliferation after addition of 6 μM Iripin-3 (**Figures 9B, F**). Therefore, Iripin-3 impairs both the viability and proliferation of CD4^+^ T cells.

### Iripin-3 Inhibits a Th1 Immune Response and Promotes Differentiation of Regulatory T Cells (Tregs) *In Vitro*

**Figure 10:**
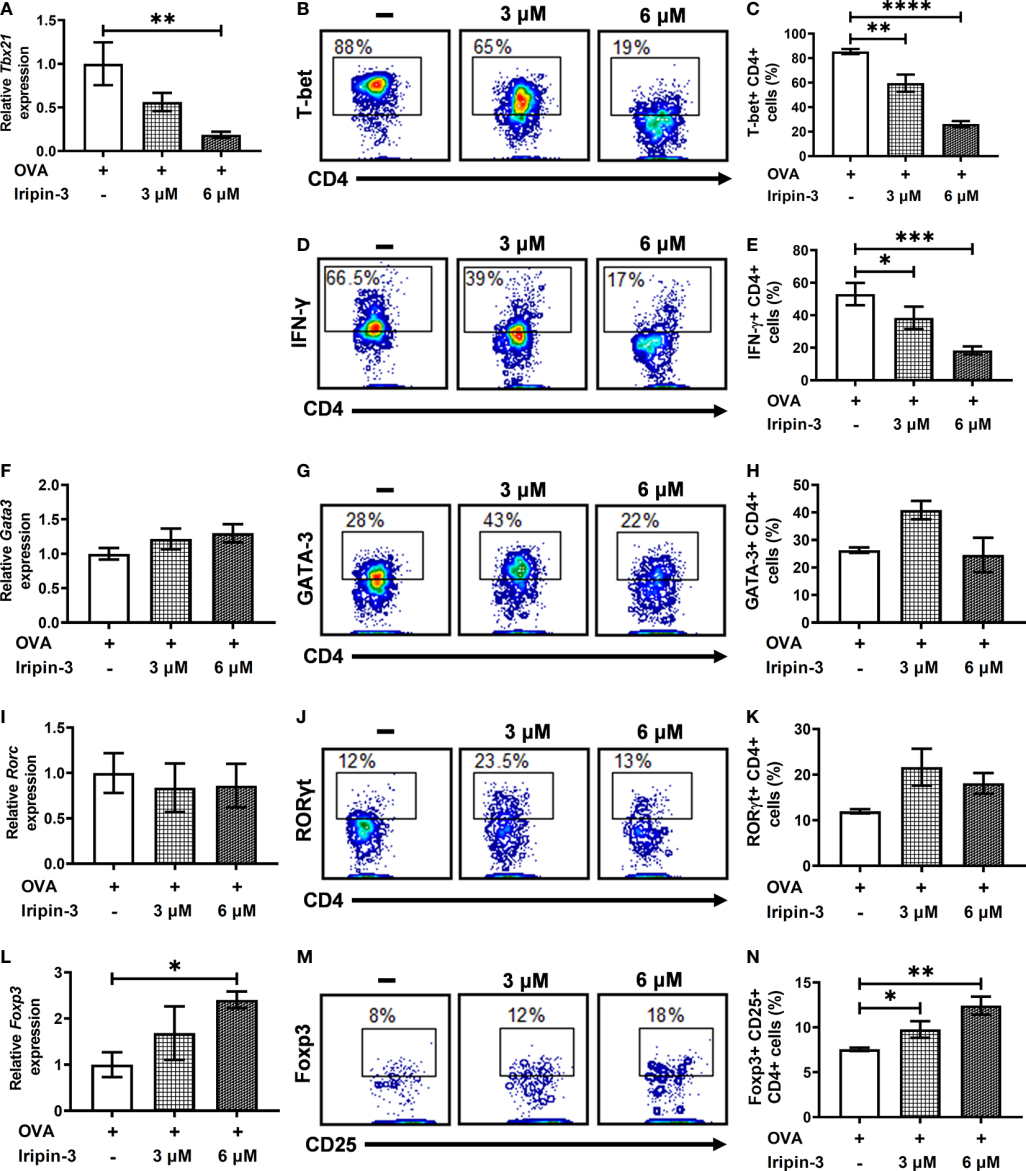
Iripin-3 alters the expression of CD4^+^ T cell transcription factors at both the mRNA and protein levels. **(A, F, I, L)** Expression of *Tbx21* **(A)**, *Gata3* **(F)**, *Rorc* **(I)**, and *Foxp3* **(L)** in CD4^+^ cells stimulated with OVA peptide for 72 h. Cells were untreated with Iripin-3 or were treated with 3 μM or 6 μM Iripin-3. Cells incubated only in the presence of OVA peptide were utilized as a calibrator during calculations of relative expression values. Data are presented as mean ± SEM of four independent experiments (* p < 0.05, ** p < 0.01). **(B, D, G, J, M)** Representative contour plots showing the proportion of OVA peptide-stimulated CD4^+^ splenocytes expressing T-bet **(B)**, IFN-γ **(D)**, GATA-3 **(G)**, RORγt **(J)** and the combination of CD25 and Foxp3 **(M)**. The cells were incubated in the absence of Iripin-3 (left) or in the presence of two different Iripin-3 concentrations: 3 μM (middle) and 6 μM (right). **(C, E, H, K, N)** The percentage of CD4^+^ T cells producing the cytokine IFN-γ **(E)** and expressing transcription factors T-bet **(C)**, GATA-3 **(H)**, RORγt **(K)**, and Foxp3 together with CD25 **(N)**. Cells were cultured in the presence of Iripin-3 (3 μM or 6 μM) and OVA peptide for 72 h. Cells incubated without Iripin-3 were used as control. Data are presented as mean ± SEM of three or four independent experiments (*p < 0.05, **p < 0.01, ***p < 0.001, ****p < 0.0001).

To examine whether Iripin-3 alters the differentiation of naïve CD4^+^ T cells into Th1, Th2, Th17, or Treg subpopulations, we evaluated the expression of transcription factors T-bet, GATA-3, RORγt, and Foxp3 in OVA peptide-stimulated CD4^+^ splenocytes by RT-qPCR and flow cytometry. T-bet, GATA-3, RORγt, and Foxp3 are considered lineage-specifying transcription factors that govern Th1, Th2, Th17, and Treg differentiation, respectively (75–79). Iripin-3 markedly and dose-dependently inhibited the expression of T-bet in CD4^+^ T cells at both the mRNA and protein levels (**Figures 10A–C**). Since T-bet controls *Ifng* transcription (76), we also tested the ability of Iripin-3 to inhibit the production of this hallmark Th1 cytokine. As with T-bet, Iripin-3 induced a pronounced and dose-dependent reduction in the percentage of CD4^+^ T cells producing IFN-γ (**Figures 10D, E**). Despite the inhibition of the Th1 immune response, we did not observe significant changes in the differentiation of T cells into Th2 or Th17 subpopulations (**Figures 10F–K**). GATA-3 expression was slightly increased only in CD4^+^ T cells treated with 3 μM Iripin-3 (**Figures 10G, H**). Similarly, both Iripin-3 concentrations induced only a small and non-significant increase in the percentage of CD4^+^ T cells expressing RORγt (**Figures 10J, K**). Finally, Iripin-3 moderately stimulated the expression of Foxp3 at both the mRNA and protein levels (**Figures 10L–N**). Therefore, Iripin-3 might induce the differentiation of Tregs in addition to inhibiting Th1 cell development.

### Iripin-3 Is Not Essential for Feeding Success of *I. ricinus* Nymphs

Since *iripin-3* expression is induced in nymphs in response to blood feeding, we decided to assess the role of this serpin in the blood-feeding process by silencing *iripin-3* expression in nymphs via RNA interference. *Iripin-3* expression in *iripin-3* dsRNA-treated ticks was 34% when compared to *gfp* dsRNA-treated ticks (data not shown), suggesting that the knockdown of the target gene was successful. Despite diminished *iripin-3* expression, the time course of blood feeding and overall feeding success (i.e. the number of nymphs that reached full engorgement) did not significantly differ between control ticks and *iripin-3* dsRNA-treated ticks (**Supplementary Table 4**). The weight of fully engorged nymphs was not significantly affected by *iripin-3* silencing as well (**Supplementary Table 4**). Therefore, we can conclude that the deficiency of Iripin-3 alone is not sufficient to impair the blood meal acquisition and processing by nymphal *I. ricinus* ticks.

## Discussion

Tick saliva contains hundreds to thousands of proteins from diverse protein families (80). These salivary proteins are differentially expressed over the course of blood feeding and enable ticks to feed to repletion by maintaining blood fluidity and suppressing host defense responses (80). Serpins form one of four serine protease inhibitor families that have been discovered in ticks (72). Serpins are particularly intriguing to study, not only due to their unique trapping inhibitory mechanism but also because they regulate a variety of physiological processes in many organisms. The functional diversity of the serpin superfamily is exemplified by the widely studied human serpins, which have been shown to regulate blood pressure, transport hormones, and control blood coagulation, fibrinolysis, angiogenesis, programmed cell death, inflammation, or complement activation (81–84). We presume that ticks employ some of their serpins to modulate host defenses, as evidenced by several tick serpins with anti-platelet, anti-coagulant, anti-inflammatory, and/or immunomodulatory properties that have been shown to be secreted via saliva into the host (34–37, 72).

Here we determined the structure and partially deciphered the function of *Ixodes ricinus* serpin Iripin-3 by using several *in vitro* models. The size (377 amino acids), molecular weight (42 kDa), and 3D structure of Iripin-3, consisting of three β-sheets, ten α-helices, and a cleaved RCL, correspond to the structural parameters of typical serpins (18, 20, 71). *Iripin-3* expression was induced by blood feeding in both nymphs and adult females, suggesting Iripin-3 contributes to feeding success in both developmental stages. Of the three organs of adult ticks, the highest levels of *iripin-3* transcript were detected in the salivary glands. The presence of Iripin-3 protein in the saliva of partially engorged adults was confirmed by immunodetection. Thus, we can assume that Iripin-3 is secreted via saliva into the tick attachment site where it interferes with host anti-tick defenses. Statistically significant increase of *iripin-3* expression in response to blood feeding occurred not only in the salivary glands but also in the ovaries of adult ticks, which indicates that Iripin-3 might be somehow involved in the reproductive process. The role of serpins in tick reproduction has been evidenced recently by *Rhipicephalus haemaphysaloides* serpin RHS-8, the knockdown of which impaired oocyte maturation due to the inability of oocytes to uptake adequate amount of vitellogenin (45).

The presence of the basic amino acid residue arginine at the P1 site of the Iripin-3 RCL indicates that Iripin-3 might inhibit trypsin-like rather than chymotrypsin-like or elastase-like serine proteases (69, 85). Indeed, out of 17 selected serine proteases, Iripin-3 most potently inhibited trypsin-like serine proteases kallikrein and matriptase and exhibited weaker inhibitory activity against trypsin, thrombin, plasmin, and factor VIIa. Kallikrein participates in the activation of the intrinsic blood coagulation pathway, promotes fibrinolysis, and is also responsible for the release of the potent inflammatory mediator bradykinin, which further induces vasodilation, increases vascular permeability, and evokes pain and itch (86, 87). Matriptase is a type II transmembrane serine protease that is primarily expressed in epithelial cells and is essential for the maintenance of skin barrier function (88). Moreover, matriptase seems to be involved in cutaneous wound healing (89, 90) and might contribute to the amplification and perpetuation of the inflammatory response through the activation of protease-activated receptor-2 (PAR-2) (91). Therefore, we speculate that Iripin-3-mediated inhibition of kallikrein and matriptase contributes to tick feeding success by suppressing the inflammatory response and consequent itch and pain and by impairing wound healing.

A phylogenetic analysis of 27 functionally characterized tick serpins revealed a close phylogenetic relationship between Iripin-3 and *I. scapularis* serpin IxscS-1E1. Both serpins possess arginine at the P1 site and inhibit trypsin and thrombin (30). However, while IxscS-1E1 prolonged plasma clotting time in aPTT and TT assays and had no effect on blood clot formation in the PT assay (30), Iripin-3 inhibited only the extrinsic coagulation pathway. This indicates that the Iripin-3-mediated inhibition of kallikrein and thrombin was not sufficient to significantly impair the intrinsic and common coagulation pathways. Other blood clotting factors (XIIa, XIa, Xa) involved in the intrinsic and common pathways were not markedly inhibited by Iripin-3. Several tick serpins are capable of inhibiting the common (and perhaps intrinsic) pathway of blood coagulation (28–31, 41, 92); however, none have shown any effect on the extrinsic coagulation pathway. The extrinsic coagulation pathway is initiated by damage to a blood vessel and subsequent formation of a FVIIa/tissue factor (TF) complex, which further activates factor X (93). In view of the fact that Iripin-3 exhibited weak inhibitory activity only in the PT test and not in the aPTT test or TT test, we hypothesized that it might target either FVIIa or TF, since these two proteins are the only unique components of the extrinsic pathway. FVIIa seemed to be a more likely target for Iripin-3 given that it is a serine protease (94), and some human serpins, such as antithrombin III or protein C inhibitor, have been shown to inhibit the proteolytic activity of FVIIa (95–97). In our hands, Iripin-3 did not form a covalent complex with FVIIa either in the absence or in the presence of TF. However, the proteolytic activity of FVIIa was reduced by approximately 30% in the presence of 400 nM Iripin-3 in the kinetic enzyme-substrate assay. Therefore, the prolongation of blood clot formation in the PT assay might be caused by the non-canonical inhibition of FVIIa by Iripin-3. Alternatively, a possible interaction between Iripin-3 and TF could also prevent FVIIa/TF complex formation, leading to a lower rate of FXa generation and inhibition of blood coagulation.

In addition to the inhibition of blood coagulation, Iripin-3 displayed anti-inflammatory activity *in vitro*, since it significantly and dose-dependently attenuated the production of pro-inflammatory cytokine IL-6 by LPS-stimulated bone marrow-derived macrophages. The decreased IL-6 production was probably caused by the inhibition of *Il6* transcription and not by reduced viability of macrophages, since the metabolic activity of macrophages remained unchanged in the presence of Iripin-3. Several tick serpins have been shown to inhibit IL-6 transcription and secretion (37–39, 74, 98), which can occur as a result of serpin-mediated inhibition of proteases such as cathepsin G and cathepsin B (37). However, the inhibition of pro-inflammatory cytokine production does not have to be dependent on serpin anti-protease activity because some serpins, like Iris and α-1-antitrypsin, can alter pro-inflammatory cytokine production by binding to immune cells via exosites (98, 99). An inflammatory environment with reduced IL-6 might favor differentiation of Tregs (100–102). Splenocytes, incubated in the presence of Iripin-3 for 72 h, increased the expression of Treg-specific transcription factor Foxp3 (77, 78), suggesting that Iripin-3 indeed induces the differentiation of naïve CD4^+^ T cells into anti-inflammatory Tregs. Tregs would facilitate the suppression of the host immune response (103), which would be beneficial for feeding ticks. There is scarce evidence that tick saliva induces Treg differentiation (104, 105). The results of our *in vitro* assay indicate that salivary serpins could contribute to this particular activity of tick saliva.

Besides the reduction in IL-6 production and increase in Foxp3 expression, Iripin-3 caused a pronounced, dose-dependent decrease in B and T cell viability *in vitro*. This effect appears to be B and T cell-specific since macrophage and dendritic cell survival was not affected by Iripin-3 and the viability of LPS-stimulated neutrophils was slightly impaired only at the highest (6 μM) concentration of Iripin-3. Serpins usually protect cells from dying by reducing the proteolytic activity of enzymes (such as granzymes and caspases) involved in programmed cell death (106). However, certain serpins, e.g., kallikrein-binding protein, pigment epithelium-derived factor, or maspin, induce apoptosis of endothelial cells and some cancer cells through distinct mechanisms such as the activation of the Fas/FasL/caspase-8 signaling pathway or the permeabilization of the outer mitochondrial membrane followed by a loss of transmembrane potential (107–111). Active caspase-3 levels were only slightly and non-significantly increased in Iripin-3-treated splenocytes. Therefore, the induction of caspase-dependent apoptosis was not the main cause of impaired splenocyte viability. Various forms of caspase-independent cell death have been described such as autophagy, paraptosis, necroptosis, or necrosis (112, 113). Elucidation of the exact mechanism behind the extensive splenocyte death in the presence of Iripin-3 is, however, beyond the scope of this paper.

*I. ricinus* saliva and salivary gland extracts inhibit T cell proliferation and suppress Th1 cell differentiation while simultaneously augmenting the Th2 immune response (114–117). Iripin-3 might contribute to this immunomodulatory effect of saliva, since in our *in vitro* assays it inhibited CD4^+^ T lymphocyte proliferation and impaired the differentiation of naïve CD4^+^ T cells into Th1 cells. Impaired Th1 cell generation was evidenced by decreased expression of the Th1 lineage-specifying transcription factor T-bet and a reduced percentage of CD4^+^ T cells producing the hallmark Th1 cytokine IFN-γ. Several studies have reported inhibition of splenocyte and peripheral blood mononuclear cell proliferation in the presence of tick serpins (35, 37, 38, 40). Interestingly, the inhibition of mitosis observed in these studies was usually accompanied by decreased IFN-γ production (35, 38, 40), which might indicate, among other things, the suppression of Th1 cell differentiation. The causative mechanism of reduced cell proliferation and impaired Th1 cell differentiation in the presence of tick serpins remains unknown, but it could be associated with decreased production of certain cytokines such as IL-2, IL-12, and IFN-γ. In the case of Iripin-3, there might be a connection between the inhibition of cell proliferation and impaired viability of splenocytes, i.e., the mechanism behind B and T cell death could be also responsible for the suppression of CD4^+^ T cell division. Iripin-3-mediated differentiation of naïve CD4^+^ T cells into Tregs might also contribute to the reduction in CD4^+^ T cell proliferation, since Tregs can inhibit cell multiplication by various mechanisms including the production of immunosuppressive cytokines TGF-β and IL-35, consumption of IL-2, and conversion of ATP to adenosine (103, 118).

It is worth mentioning that the Iripin-3 concentrations used in *in vitro* experiments (3 μM and 6 μM) are probably higher than the amount of Iripin-3 at the tick feeding site. This fact, however, does not make the anticoagulant, ant-inflammatory and immunomodulatory activities of Iripin-3 observed *in vitro* physiologically irrelevant. Tick saliva is a complex mixture of proteins from the same or different protein families, and some of these salivary proteins can share the same function (119). Therefore, even a low concentration of one tick protein may be sufficient to achieve a desired effect at the tick attachment site if this protein acts in concert with other tick proteins (119). For instance, the ability of *I. ricinus* saliva to inhibit CD4^+^ T cell proliferation is probably a result of combined action of more proteins with anti-proliferative properties, such as the serpins Iripin-3 and Iris, the cystatin Iristatin and the Kunitz domain-containing protein IrSPI (38, 120, 121). That *I. ricinus* saliva may contain other proteins possessing Iripin-3-like activities was demonstrated by the RNA interference experiment. *Iripin-3* knockdown did not significantly affect the overall feeding success, time course of blood feeding and weight of fully engorged nymphs, which indicates that other similarly acting salivary proteins might compensate for the loss of *iripin-3* expression.

It is also important to note that native Iripin-3 is most likely glycosylated. However, recombinant Iripin-3 was prepared in an *E. coli* expression system, and therefore it lacks glycosylation. Glycosylation has been shown to reduce the propensity of serpins for polymerization (122) and increase the stability and half-life of circulating serpins by conferring resistance to proteolytic degradation (123, 124). The impact of glycosylation on the biological function of serpins is less clear. Recombinant Iripin-3 inhibited the proteolytic activity of some serine proteases, suggesting that its functions dependent on anti-protease activity (like anticoagulant properties) may not be affected by missing glycosylation. However, the absence of glycosylation might have an impact on anti-inflammatory and immunomodulatory activities of Iripin-3 mediated by its binding to cell surfaces and soluble immune mediators. For example, only glycosylated, but not non-glycosylated, α-1-antitrypsin was capable of binding IL-8, thus inhibiting IL-8-CXCR1 interaction (125).

## Conclusion

To conclude, Iripin-3 is a pluripotent salivary protein secreted by *I. ricinus* ticks via saliva into the feeding site, where it might suppress various aspects of host anti-tick defenses. The attenuation of IL-6 production, suppression of CD4^+^ T cell proliferation, and inhibition of Th1 immune responses have also been observed with other tick serpins and are consistent with the previously reported immunomodulatory effects of *I. ricinus* saliva and salivary gland extracts (114–117). On the other hand, our study is the first to describe the inhibition of the extrinsic pathway of blood coagulation, impaired B and T cell survival, and the induction of Treg differentiation by a tick serpin. The pluripotency and redundancy in Iripin-3 functions are consistent with the theory about the importance of these protein features for successful tick feeding (119). Although several distinct *in vitro* activities of Iripin-3 were observed in this study, their physiological relevance, mechanisms behind them and potential of Iripin-3 to be a candidate for drug or vaccine development remain to be determined. Therefore, further *in vivo* experiments and mechanistic studies are needed to validate and elucidate the Iripin-3 functions described in this work.

## Data Availability Statement

The data sets presented in this study can be found in online repositories. The names of the repository/repositories and accession number(s) can be found in the article/**Supplementary Material**.

## Ethics Statement

All animal experiments were performed in accordance with the Animal Protection Law of the Czech Republic No. 246/1992 Sb. (ethics approval no. 34/2018) and protocols approved by the Ministry of Education, Youth and Sports of the Czech Republic (protocol no. 19085/2015-3) and the responsible committee of the IP BC CAS. Pathogen-free *I. ricinus* ticks were obtained from the tick colony maintained at the IP BC CAS.

## Author Contributions

AC designed and performed experiments, analyzed data, and wrote the manuscript. JK, ZB, BK, LAM, HL, TP, ME, and IKS designed and performed experiments and analyzed data. MK edited the manuscript. JC directed the study, designed experiments, analyzed data, and edited the manuscript. All authors contributed to the article and approved the submitted version.

## Funding

This work was financed by the Grant Agency of the Czech Republic (grant 19-14704Y to JC and grant 19-382 07247S to MK) and by the Grant Agency of the University of South Bohemia (grant 105/2019/P to AC). It was also supported by ERDF no. CZ.02.1.01/0.0/0.0/15_003/0000441 to IKS.

## Conflict of Interest

The authors declare that the research was conducted in the absence of any commercial or financial relationships that could be construed as a potential conflict of interest.
