# Supplementary material for: Iripin-3, a New Salivary Protein Isolated From Ixodes ricinus Ticks, Displays Immunomodulatory and Anti-Hemostatic Properties In Vitro
Source: Front Immunol. 2021 Mar 1;12:626200. doi: 10.3389/fimmu.2021.626200 (PMC7957079; doi:10.3389/fimmu.2021.626200)
Supplement: Supplementary file 1 [file DataSheet_1.docx]

Supplementary Material

Materials and methods

**Production of recombinant Iripin-3**

A full-length Iripin-3 sequence was obtained during a salivary gland transcriptome project (1) and was submitted to GenBank under accession number GADI01004776.1. The Iripin-3 nucleotide sequence without a signal peptide and with an ATG codon inserted into its 5’-terminus was cloned into the pET-17b vector (Novagen, MilliporeSigma, Burlington, MA), and the resulting plasmid was transformed into BL21(DE3)pLysS chemically competent E. coli cells (Thermo Fisher Scientific, Waltham, MA). Cells were grown in LB medium containing ampicillin (100 μg/ml) and chloramphenicol (34 μg/ml), and when the OD600 of the culture reached approximately 0.7, isopropyl β-D-1-thiogalactopyranoside (IPTG, 0.5 mM) was added to induce gene expression. Cells were harvested after 3.5 h incubation in the presence of IPTG and the cell pellet was resuspended in 20 mM Tris-HCl (pH 8). Cell disruption by sonication in inclusion bodies isolation buffer (20 mM Tris-HCl, 1% Triton X-100, pH 8) and repeated washing with 20 mM Tris-HCl (pH 8) resulted in a pellet that contained mainly inclusion bodies of insoluble recombinant Iripin-3. The inclusion bodies were dissolved by stirring in 6 M guanidine hydrochloride (pH 8) and 10 mM dithiotreitol for 1 h at room temperature. Following centrifugation, the supernatant, which contained denatured Iripin-3 released from the inclusion bodies, was diluted 150-fold in refolding buffer (20 mM Tris-HCl, 150 mM NaCl, pH 8), and the mixture was incubated overnight at 4°C. The precipitated protein was removed by filtration through filter paper and Steritop-GP (MilliporeSigma) and concentrated with a stirred chamber concentrator (MilliporeSigma). Properly refolded Iripin-3 was then dialyzed against 20 mM Tris-HCl (pH 8) and purified on a HiLoad 26/60 Superdex 200 pg gel filtration column (Cytiva) and a Mono Q column (Cytiva) with the 0-1 M gradient of NaCl. Endotoxin was removed by the company ARVYS Proteins, Inc. (Trumbull, CT) via a detergent-based method.

**Crystallization**

Crystallization experiments were performed in Swissci 96-well 2-drop MRC crystallization plates (Molecular Dimensions Ltd., Sheffield, UK) using the sitting-drop vapor diffusion technique and OryxNano crystallization robot (Douglas Instruments Ltd., Hungerford, UK). A suitable Iripin-3 concentration (1.88 mg/ml) was determined by the PCT Pre-Crystallization Test (Hampton Research, Aliso Viejo, CA). Iripin-3 crystals were grown with the precipitant composed of 0.2 M potassium thiocyanate, 0.1 M sodium cacodylate, and 8% w/v γ-polyglutamic acid, pH 6.5 at 21°C. The protein-to-precipitant solution ratios 2:1 (2 μl:1 μl) or 1:1 (1 μl:1 μl) were equilibrated against 50 μl of reservoir solution.

**X-ray data collection and structure determination**

Freshly grown crystals were flash frozen in a liquid nitrogen stream without additional cryoprotection, and X-ray diffraction data were collected at the BESSY II electron storage ring on the beamline BL14.1 operated by the Helmholtz-Zentrum Berlin (2). Data were processed using the XDS Program Package (3) with the XDSAPP graphical user interface (4). The best diffracting crystal exhibited symmetry of the *P*6_2_22 space group and contained one molecule in the asymmetric unit. The structure of Iripin-3 was solved by the molecular replacement method using MOLREP (5). Of all the structures deposited in the PDB, IRS-2 (PDB code 3NDA) (6) displayed the highest sequence identity (56%) to Iripin-3 and was therefore used as a search model. The Iripin-3 structure was refined with the program REFMAC5 (7) from the CCP4 suite (8) and manually rebuilt in Coot (9). MolProbity (10) and wwPDB (11) were used for final qualitative validation of the model. Figures of the Iripin-3 structure were made using the PyMOL Molecular Graphic System (Schrödinger, LLC, New York, NY). Atomic coordinates were deposited in the PDB under accession code 7AHP. Data collection, processing, and refinement statistics are summarized in **Supplementary Table 1**.

**Presence of Iripin-3 in tick saliva (ELISA)**

Each well of a Corning 96-well microplate was coated overnight at 4°C with 50 μl of tick saliva diluted in coating buffer (15 mM Na_2_CO_3_, 35 mM NaHCO_3_, pH 9.6) to a final concentration of 10 μg/ml. The unoccupied binding sites of the plate were blocked by the addition of 300 μl/well of blocking buffer (5% precolostral calf serum in PBS) for 1 h at 37 °C. After washing the plate three times with wash buffer (0.05% Tween 20 in PBS), 50 μl of pre-immune serum or antiserum against Iripin-3, pre-diluted 1:1000 with 2% precolostral calf serum in PBS, was added and incubated for 1 h at 37°C. Another three washes with wash buffer were followed by the addition of 100 μl/well of peroxidase-conjugated goat antibody recognizing rabbit immunoglobulin G (Sigma Aldrich, St Louis, MO). The antibody was pre-diluted 1:1000 with 2% precolostral calf serum in PBS and incubated for 1 h at 37°C. At the end of incubation, the plate was again washed three times with wash buffer, and then 100 μl of a substrate solution (51.4 mM Na_2_HPO_4_ · 12 H_2_O, 24.3 mM C_6_H_8_O_7_ · H_2_O, 3.7 mM *o*-phenylenediamine, and 0.012% H_2_O_2_, pH 5) was applied to each well. The enzymatic reaction was stopped by the addition of 2 M H_2_SO_4_, and the optical density was measured at 490 nm on Synergy H1 microplate reader (BioTek Instruments, Inc., Winooski, VT).

**Presence of Iripin-3 in tick saliva (Western blot)**

Tick saliva (10 μg) and Iripin-3 (1 ng or 10 ng) were subjected to SDS-PAGE using a 10% gel, and the separated proteins were transferred to polyvinylidene difluoride (PVDF) membranes (Thermo Fisher Scientific, Waltham, MA). Following blocking in Tris-buffered saline containing 5% fat-free milk and 0.1% Tween 20 for 1 h at room temperature, the blots were incubated overnight at 4°C with pre-immune serum or antiserum against Iripin-3. Both sera were pre-diluted 1:100 in Tris-buffered saline containing 5% milk and 0.1% Tween 20. After washing, the membranes were incubated with goat anti-rabbit antibody conjugated with horseradish peroxidase (Cell Signaling Technology, Danvers, MA) for 1 h at room temperature. The secondary antibody was pre-diluted 1:2000 in the same solution as the primary antibodies. The proteins were visualized using the enhanced chemiluminescent substrate WesternBright Quantum (Advansta, San Jose, CA), and the signal was detected using a charge-coupled device (CCD) imaging system (Uvitec Ltd., Cambridge, UK).

**Inhibition of serine proteases**

First, the formation of SDS- and heat-stable complexes between Iripin-3 and selected serine proteases (kallikrein, matriptase, plasmin, thrombin, trypsin, factor VIIa) was tested. All enzymes used were of human origin. Kallikrein and thrombin were purchased from Sigma Aldrich, matriptase and trypsin were purchased from R&D Systems (Minneapolis, MI), and plasmin and factor VIIa were obtained from Haematologic Technologies, Inc. (Essex Junction, VT). Iripin-3 and proteases were diluted in assay buffer corresponding to each protease (described below), and then each of the six serine proteases was incubated with Iripin-3 at equimolar concentrations (1 μM) for 1 h at room temperature. Factor VIIa was incubated with Iripin-3 in the absence or presence of human tissue factor (1 μM, BioLegend, San Diego, CA). The 1 h incubation was followed by the addition of NuPAGE LDS Sample Buffer (Thermo Fisher Scientific) together with dithiotreitol and boiling of samples for 10 min. Finally, samples were analyzed by SDS-PAGE and protein bands were visualized by silver staining.

Second, second-order rate constants of protease inhibition were measured by a discontinuous method under pseudo first-order conditions using at least a 50-fold molar excess of Iripin-3 over serine proteases. Reactions were incubated at room temperature and were stopped at each time point by the addition of the fluorogenic substrate appropriate for the protease used. The slope of the linear part of fluorescence increase over time gave the residual protease activity at each time point. The apparent (observed) first-order rate constant k_obs_ was calculated from the slope of the plot of the natural log of residual protease activity against time. K_obs_ was measured for six different Iripin-3 concentrations and plotted against the serpin concentration. The slope of the line of best fit gave an estimate of the second-order rate constant k_2_. The assay buffer was 20 mM Tris, 150 mM NaCl, 0.02% Triton X-100, pH 8.5 for kallikrein and plasmin; 50 mM Tris, 50 mM NaCl, 0.01% Tween 20, pH 9.0 for matriptase; 20 mM Tris, 150 mM NaCl, 0.01% Triton X-100, 5 mM CaCl_2_, 0.1% polyethylene glycol 6000, pH 8.0 for thrombin and factor VIIa; and 50 mM Tris, 150 mM NaCl, 10 mM CaCl_2_, 0.05% Brij 35, pH 7.5 for trypsin. The fluorogenic substrates used were Z-FR-AMC for kallikrein, Boc-QAR-AMC for matriptase, and Boc-VPR-AMC for trypsin and thrombin. All substrates were used at 250 μM final concentration. Kallikrein, matriptase, thrombin, and trypsin were used at 200 pM, 500 pM, 20 pM, and 2 pM final concentrations, respectively.

The HADDOCK2.2 web server (12) was used for docking of the peptide consisting of Iripin-3 RCL residues P4-P4' inside the active site of four proteases (trypsin, thrombin, kallikrein and matriptase). The Iripin-3 structure was modified, thus alternative conformations and ligands were removed. The visualization of the docking results together with electrostatic potential depiction and polar contact analysis were made in the PyMOL Molecular Graphics System (Schrödinger, LLC). Tertiary structures of proteases were retrieved from the Protein Data Bank. PDB accession codes are 5TJX for kallikrein, 1EAX for matriptase, 3U69 for thrombin and 1H4W for trypsin.

**Pro-inflammatory cytokine expression in bone marrow-derived macrophages (RT-qPCR)**

Total RNA was isolated from macrophages using TRI Reagent (Molecular Research Center, Inc., Cincinnati, OH) according to the manufacturer's instructions. Extracted RNA (500 ng) was treated with DNase I (Thermo Fisher Scientific) and reverse transcribed into cDNA using M-MLV Reverse Transcriptase (Thermo Fisher Scientific) as detailed in the manufacturer’s protocol. The resulting cDNA mixed with Maxima SYBR Green/ROX qPCR Master Mix (Thermo Fisher Scientific) and gene-specific primers were used for the analysis of *Tnf*, *Il6*, and *Il1b* expression in the QuantStudio 6 thermal cycler (Thermo Fisher Scientific)**.** Cycling conditions were 95°C for 10 min followed by 40 cycles of 95°C for 15 s and 60°C for 60 s. Relative gene expression was calculated using the delta-delta Ct method, since the amplification efficiencies of target genes and a reference gene (*Gapdh*) were approximately equal (13). Nucleotide sequences of forward and reverse primers as well as amplicon lengths are provided in **Supplementary Table 3**.

**Viability of macrophages, dendritic cells, and neutrophils**

Dendritic cells and macrophages were obtained as described before (14). Briefly, dendritic cells were derived from bone marrow cells of a C57BL/6N mouse by incubating the cells for 8 days in the presence of 20 ng/ml of GM-CSF (Sigma Aldrich). Macrophages were obtained from bone marrow cells by 7-day incubation in the presence of L929 cell-conditioned medium. Neutrophils were isolated from bone marrow cells by magnetic separation using a Neutrophil Isolation Kit (Miltenyi Biotec, Bergisch Gladbach, Germany). Dendritic cells, macrophages, and neutrophils were resuspended in RPMI 1640 medium with stable glutamine (Biosera, Kansas City, MO) supplemented with 10% heat-inactivated FBS (Biosera), 50 μM 2-mercaptoethanol (Sigma Aldrich), 100 U/ml penicillin G (Biosera), and 100 μg/ml streptomycin (Biosera) and then were treated with four different concentrations of Iripin-3 for 1 h at 37°C and 5% CO_2_. Subsequently, macrophages and neutrophils were stimulated by the addition of LPS (100 ng/ml, Sigma Aldrich, *E. coli* serotype O111:B4). Dendritic cells were left unstimulated. After incubating the cells for 20 h at 37°C and 5% CO_2_, alamarBlue HS Cell Viability Reagent (Thermo Fisher Scientific) was added. The fluorescence intensity was measured on Synergy H1 microplate reader (BioTek Instruments, Inc.; excitation 550 nm; emission 590 nm) 4 h (a 24h incubation period) and 28 h (a 48h incubation period) following alamarBlue addition.

***Iripin-3* knockdown in *I. ricinus* nymphs by the RNA interference technique**

A 550-bp fragment of the *iripin-3* gene was amplified from *I. ricinus* cDNA using primers 5´-ATTCTA GAGTCATTCTTTAACGGTGGCG-3´ and 5´-ATGGGCCCAAAAAGGATGGCGTTTGTGC-3´ that contained restriction sites XbaI and ApaI. The amplified fragment was cloned into the pLL10 vector with two T7 promoters in reverse orientations (15). Double-stranded RNA of *iripin-3* and green fluorescent protein (*gfp*) were synthesized using the MEGAscript T7 Transcription Kit (Thermo Fisher Scientific) as described previously (16). The dsRNA (32 nl; 3 µg/µl) was injected into the haemocoel of *I. ricinus* nymphs using Nanoinject II (Drummond Scientific Company, Broomall, PA). After a 3-day rest in a humid chamber at room temperature, ticks were fed on C3H/HeN mice (16-20 nymphs per mouse) until full engorgement. Three tick feeding parameters were evaluated - feeding duration, overall feeding success and engorgement weight. The level of gene knockdown was checked by RT-qPCR in an independent feeding experiment.

**Supplementary Table 1. X-ray data collection, processing and refinement statistics.**

| **Data collection** | | |
| --- | --- | --- |
| X-ray source | | BL14.1, BESSY II, Germany |
| Wavelength (Å) | | 0.9184 |
| Detector | | PILATUS 6M |
| Crystal-detector distance (mm) | | 222.687 |
| Rotation range per image (°) | | 0.1 |
| Total rotation range (°) | | 240 |
| Exposure time per image (s) | | 0.25 |
| Resolution range (Å) | | 48.32-1.95 (2.07-1.95) |
| Space group | | *P*6_2_22 |
| Unit-cell dimensions: a, b, c (Å) | | 132.94, 132.94, 88.89 |
| Unit-cell dimensions: α, β, γ (°) | | 90.0, 90.0, 120.0 |
| Mosaicity (°) | | 0.135 |
| Total number of reflections | | 889221 (130341) |
| Number of unique reflections | | 34278 (5397) |
| Multiplicity | | 25.94 (24.15) |
| Average I/σ(I) | | 13.45 (2.45) |
| Completeness (%) | | 99.9 (99.4) |
| CC ½ | | 99.8 (82.6) |
| R_meas_ (%)^a^ | | 25.9 (157.3) |
| Overall B factor from Wilson plot (Å^2^) | | 29.14 |
| **Refinement** | | |
| Resolution range (Å) | | 48.32-1.95 (2.07-1.95) |
| Number of reflections in working set | | 32559 (2340) |
| Final R value (%)^b^ / Final R_free_ value (%)^c^ | | 19.16 / 22.28 |
| Mean B value (Å) | | 24.26 |
| Number of atoms in the asymmetric unit | | |
| Protein | | 2881 |
| Ligand-Tris ion | | 3 |
| Water | | 243 |
| Total | | 3148 |
| Root-mean-square deviations | | |
| Bonds (Å) | | 0.015 |
| Angles (°) | | 1.709 |
| Average B factors (Å^2^) Overall | 24.264 | |
| Ramachandran plot | | |
| Most favored (%) | | 98.90 |
| Allowed (%) | | 100.00 |
| PDB code | | 7AHP |

The data in parentheses refer to the highest resolution shell.

^a^ R_meas_ = (|I_hkl_ - 〈I〉|)/I_hkl_, where the average intensity 〈I〉 is taken over all symmetry equivalent measurements, and I_hkl_ is the measured intensity for any given reflection.

^b^ R value = ||F_o_| - |F_c_||/|F_o_|, where F_o_ and F_c_ are the observed and calculated structure factors, respectively.

^c^ R_free_ is equivalent to R value but is calculated for 5% of the reflections chosen at random and omitted from the refinement process.

**Supplementary Table 2. GenBank accession numbers of serpins used in the phylogenetic analysis.**

| **Serpin** | **Species** | **GenBank accession number** | **Reference** |
| --- | --- | --- | --- |
| A1AT | *Homo sapiens* | AAB59495.1 | (17) |
| AamS6 | *Amblyomma americanum* | ABS87358.1 | (18) |
| AAS19 |  | JAI08902.1 | (19) |
| AAS27 |  | JAI08961.1 |  |
| AAS41^a^ |  | JAI08957.1 | (19,20) |
| AAS46^a^ |  | JAI08784.1 |  |
| HLS1^b^ | *Haemaphysalis longicornis* | Not found | (21) |
| HLS2 |  | BAD11156.1 | (22) |
| HlSerpin-a |  | QFQ50847.1 | (23) |
| HlSerpin-b |  | QFQ50848.1 |  |
| Ipis-1 | *Ixodes persulcatus* | BAP59746.1 | (24) |
| Iripin-3 | *Ixodes ricinus* | JAA69032.1 |  |
| Iris |  | CAB55818.2 | (25) |
| IRS-2 |  | ABI94056.2 | (6) |
| IxscS-1E1 | *Ixodes scapularis* | AID54718.1 | (26) |
| RAS-1 | *Rhipicephalus appendiculatus* | AAK61375.1 | (27) |
| RAS-2 |  | AAK61376.1 |  |
| RAS-3 |  | AAK61377.1 |  |
| RAS-4 |  | AAK61378.1 |  |
| RHS-1 | *Rhipicephalus haemaphysaloides* | AFX65224.1 | (28) |
| RHS-2 |  | AFX65225.1 |  |
| RHS8 |  | QHU78941.1 | (29) |
| RmS-1 | *Rhipicephalus microplus* | AHC98652.1 | (30) |
| RmS-3 |  | AHC98654.1 |  |
| RmS-6 |  | AHC98657.1 |  |
| RmS-15 |  | AHC98666.1 |  |
| RmS-17 |  | AHC98668.1 |  |
| rSERPIN^b^ |  | Not found | (31) |

^a^ Full-length protein sequences of AAS41 and AAS46 were obtained from the cited article (20), since GenBank contains only partial amino acid sequences of these two *A. americanum* serpins.

^b^ In the case of two tick serpins (HLS1 and rSERPIN), no accession number was found, and therefore the amino acid sequences needed for phylogenetic analysis were derived directly from the cited articles.

# Supplementary Table 3. Sequences of primers used in the study.

| **Gene** | **Species** | **Sequence** | **Amplicon length (bp)** |  |
| --- | --- | --- | --- | --- |
| **Iripin-3 expression in ticks** | | | | |
| *rps4* | *Ixodes ricinus* | Forward: 5´-GGTGAAGAAGATTGTCAAGCAGAG-3´  Reverse: 5´-TGAAGCCAGCAGGGTAGTG-3´ | 80 |  |
| *iripin-3* |  | Forward: 5´-CACAGCGGCAATTCATTTAGG-3´  Reverse: 5´-CGGTACGTCTCTTCTGAAACTC-3´ | 269 |  |
| **Pro-inflammatory cytokine expression in macrophages** | | | | |
| *Gapdh* | *Mus musculus* | Forward: 5’-TGTGTCCGTCGTGGATCTGA-3’  Reverse: 5’-TTGCTGTTGAAGTCGCAGGAG-3’ | 150 |  |
| *Il1b* |  | Forward: 5’-TGACCTGGGCTGTCCTGATG-3’  Reverse: 5’-GGTGCTCATGTCCTCATCCTG-3’ | 160 |  |
| *Il6* |  | Forward: 5’-CTGCAAGAGACTTCCATCCAG-3’  Reverse: 5’-AGTGGTATAGACAGGTCTGTTGG-3’ | 131 |  |
| *Tnf* |  | Forward: 5’-CCCCAAAGGGATGAGAAGTTC-3’  Reverse: 5’-GGCTTGTCACTCGAATTTTGAGA-3’ | 101 |  |
| **Transcription factor expression in CD4^+^ T cells** | | | | |
| *Actb* | *Mus musculus* | Forward: 5’-CTCTGGCTCCTAGCACCATGAAGA-3’  Reverse: 5’-GTAAAACGCAGCTCAGTAACAGTCCG-3’ | 200 |  |
| *Foxp3* |  | Forward: 5’-CAGCTCTGCTGGCGAAAGTG-3’  Reverse: 5’-TCGTCTGAAGGCAGAGTCAGGA-3’ | 190 |  |
| *Gapdh* |  | Forward: 5’-TGTGTCCGTCGTGGATCTGA-3’  Reverse: 5’-TTGCTGTTGAAGTCGCAGGAG-3’ | 150 |  |
| *Gata3* |  | Forward: 5’-CTCGGCCATTCGTACATGGAA-3’  Reverse: 5’-GGATACCTCTGCACCGTAGC-3’ | 134 |  |
| *Rorc* |  | Forward: 5’-ACGGCCCTGGTTCTCATCA-3’  Reverse: 5’-CCAAATTGTATTGCAGATGTTCCAC-3’ | 79 |  |
| *Tbx21* |  | Forward: 5’-TCAACCAGCACCAGACAGAGA-3’  Reverse: 5’-TCCACCAAGACCACATCCAC-3’ | 130 |  |

**Results**

**
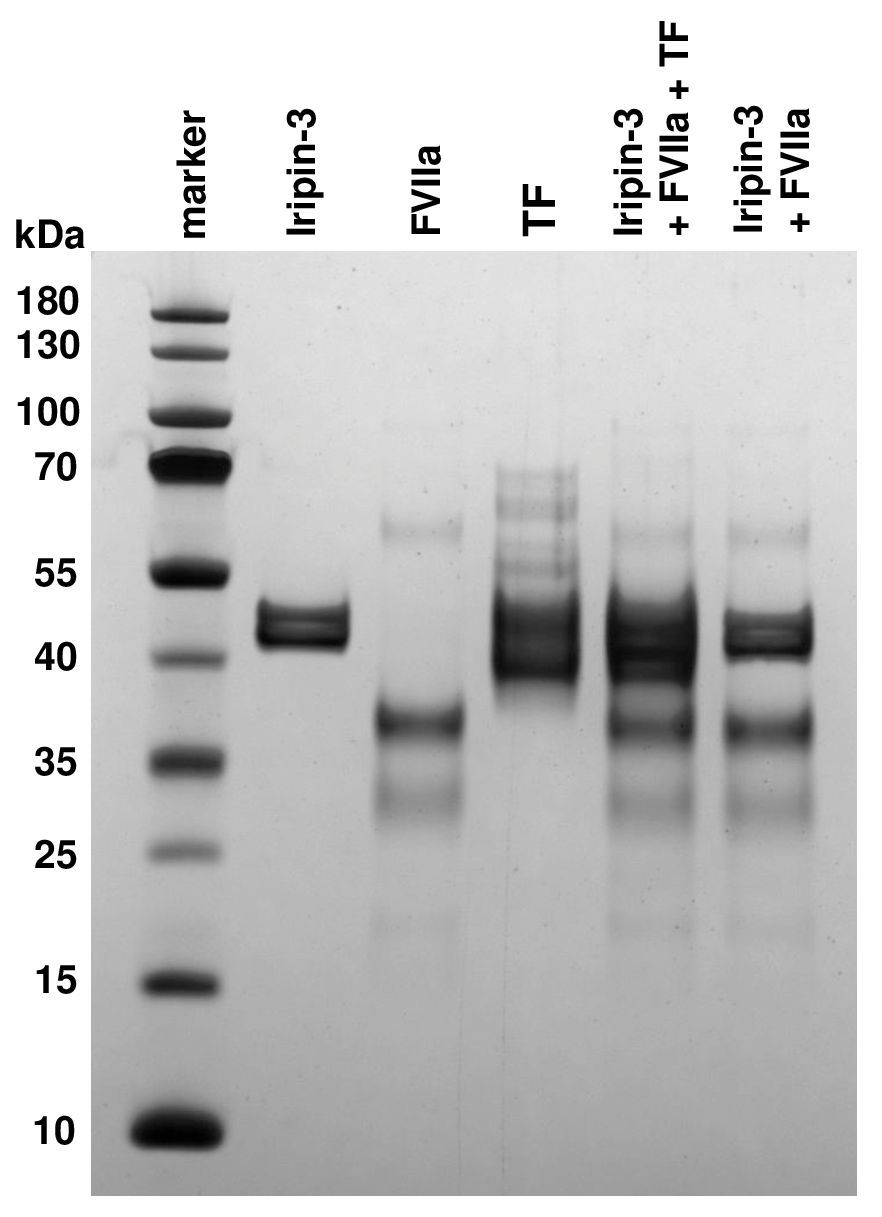
**

**Supplementary Figure 1. Iripin-3 does not form a covalent complex with activated factor VII (FVIIa).** A high molecular weight complex formation between FVIIa and Iripin-3 in the absence or presence of tissue factor (TF) was analyzed using SDS-PAGE. Proteins were resolved on 4 to 12% NuPAGE Bis-Tris gels and were visualized by silver staining.


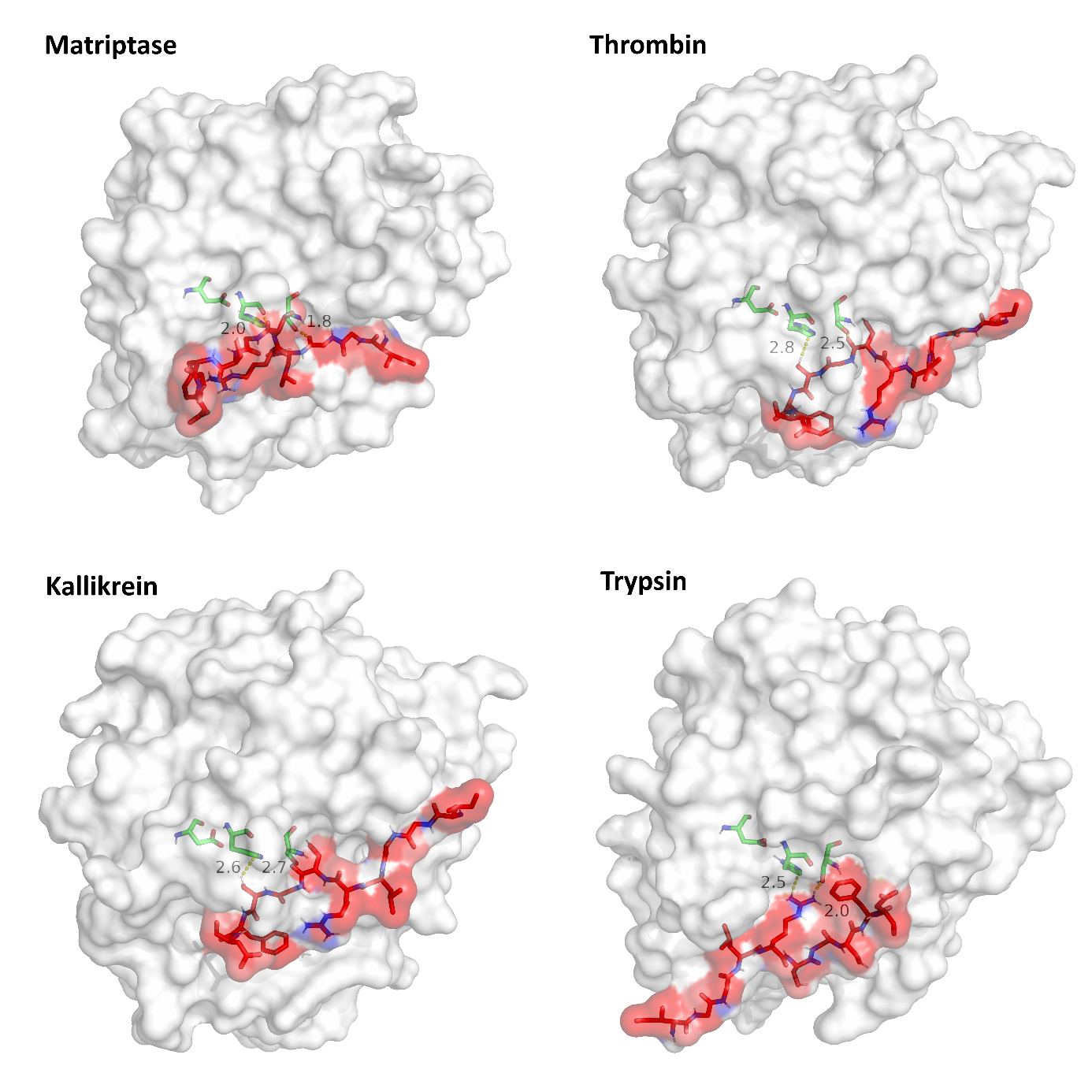


**Supplementary Figure 2. Interface analysis of the proteases, for which *k_2_* was determined, and the P4-P4' part of Iripin-3 RCL.** The surfaces of the four proteases are colored white, and the P4-P4' region of Iripin-3 RCL is represented by a cartoon (red). Predicted interactions shorter than 2.8 Å are shown with dotted lines. The catalytic triad of the proteases is depicted in green.


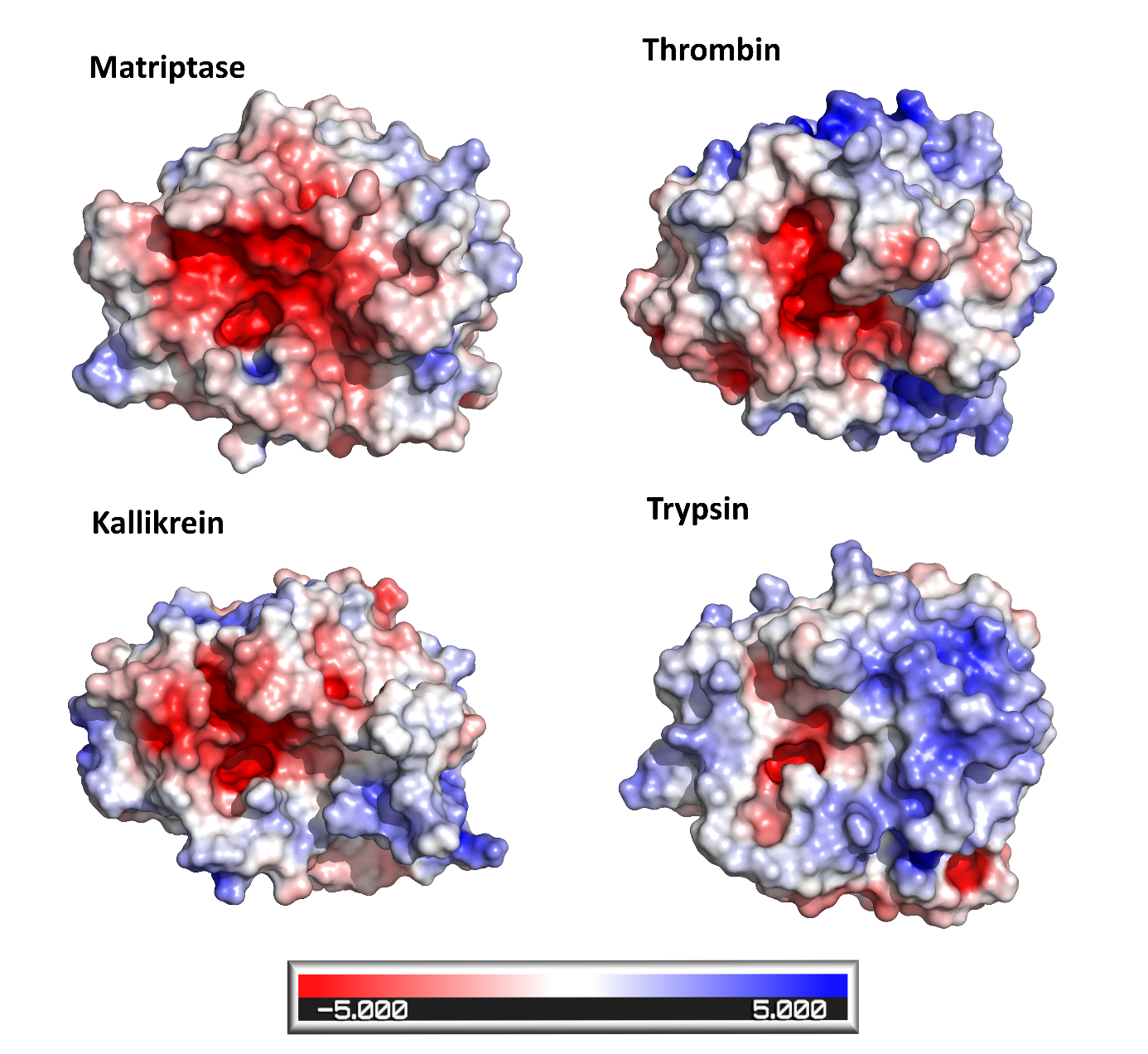


**Supplementary Figure 3. Electrostatic potential on the surfaces of matriptase, thrombin, kallikrein and trypsin.** Active sites of these serine proteases are negatively charged (red color), which enables binding of suitable substrates and/or inhibitors.

**A**

**B**

**C**

**Supplementary Figure 4. Iripin-3 does not negatively affect macrophage, dendritic cell, and neutrophil viability.** Cell viability was evaluated by their ability to reduce virtually non-fluorescent resazurin, the active ingredient of alamarBlue, to highly fluorescent resorufin. **(A, B)** The viability of unstimulated or LPS-stimulated macrophages **(A)** and unstimulated dendritic cells **(B)** after exposure to four different concentrations of Iripin-3 for either 24 h or 48 h. Macrophages and dendritic cells unexposed to Iripin-3 were used as control. **(C)** The viability of LPS-stimulated neutrophils untreated with Iripin-3 or treated with four different concentrations of Iripin-3 for 24 h. All data in **(A, B, C)** are presented as mean ± SEM. The experiment was performed only once. RFU, relative fluorescence unit.

Supplementary Table 4. The overall feeding success, time course of blood feeding and weight of fully engorged *I. ricinus* nymphs were not significantly affected by *iripin-3* silencing via RNA interference.

| **Silenced gene** | **Feeding success**^a^ | **FF < 48 h** | **FF 48-72 h**^a^ | **FF 72-96 h**^a^ | **FF ˃ 96 h**^a^ | **Weight (mg)**^b^ |
| --- | --- | --- | --- | --- | --- | --- |
| *gfp* | 92.1 % (70/76) | 0 | 30.0 % (n=21) | 61.4 % (n=43) | 8.6 % (n=6) | 3.51 ± 1.03 |
| *iripin-3* | 92.2 % (71/77) | 0 | 33.8 % (n=24) | 57.7 % (n=41) | 8.5 % (n=6) | 3.79 ± 0.92 |

FF, fully fed nymphs. Parentheses contain the number of nymphs that reached full engorgement within given time periods.

^a^ Data were analyzed by the two-tailed Fisher’s exact test.

^b^ Data, expressed as mean ± standard deviation, were analyzed by the unpaired two-tailed *t*-test.

References

1. Schwarz A, von Reumont BM, Erhart J, Chagas AC, Ribeiro JMC, Kotsyfakis M. *De novo Ixodes ricinus* salivary gland transcriptome analysis using two next-generation sequencing methodologies. *FASEB J* (2013) **27**:4745–4756. doi:10.1096/fj.13-232140

2. Gerlach M, Mueller U, Weiss MS. The MX beamlines BL14.1-3 at BESSY II. *JLSRF* (2016) **2**:47. doi:10.17815/jlsrf-2-64

3. Kabsch W. XDS. *Acta Crystallogr D Biol Crystallogr* (2010) **66**:125–132. doi:10.1107/S0907444909047337

4. Sparta KM, Krug M, Heinemann U, Mueller U, Weiss MS. XDSAPP2.0. *J Appl Cryst* (2016) **49**:1085–1092. doi:10.1107/S1600576716004416

5. Vagin A, Teplyakov A. MOLREP: an automated program for molecular replacement. *J Appl Cryst* (1997) **30**:1022–1025. doi:10.1107/S0021889897006766

6. Chmelar J, Oliveira CJ, Rezacova P, Francischetti IMB, Kovarova Z, Pejler G, Kopacek P, Ribeiro JMC, Mares M, Kopecky J, et al. A tick salivary protein targets cathepsin G and chymase and inhibits host inflammation and platelet aggregation. *Blood* (2011) **117**:736–744. doi:10.1182/blood-2010-06-293241

7. Murshudov GN, Skubák P, Lebedev AA, Pannu NS, Steiner RA, Nicholls RA, Winn MD, Long F, Vagin AA. REFMAC5 for the refinement of macromolecular crystal structures. *Acta Crystallogr D Biol Crystallogr* (2011) **67**:355–367. doi:10.1107/S0907444911001314

8. Winn MD, Ballard CC, Cowtan KD, Dodson EJ, Emsley P, Evans PR, Keegan RM, Krissinel EB, Leslie AGW, McCoy A, et al. Overview of the CCP4 suite and current developments. *Acta Crystallogr D Biol Crystallogr* (2011) **67**:235–242. doi:10.1107/S0907444910045749

9. Emsley P, Lohkamp B, Scott WG, Cowtan K. Features and development of Coot. *Acta Crystallogr D Biol Crystallogr* (2010) **66**:486–501. doi:10.1107/S0907444910007493

10. Chen VB, Arendall WB, Headd JJ, Keedy DA, Immormino RM, Kapral GJ, Murray LW, Richardson JS, Richardson DC. MolProbity: all-atom structure validation for macromolecular crystallography. *Acta Crystallogr D Biol Crystallogr* (2010) **66**:12–21. doi:10.1107/S0907444909042073

11. Gore S, Velankar S, Kleywegt GJ. Implementing an X-ray validation pipeline for the Protein Data Bank. *Acta Crystallogr D Biol Crystallogr* (2012) **68**:478–483. doi:10.1107/S0907444911050359

12. van Zundert GCP, Rodrigues JPGLM, Trellet M, Schmitz C, Kastritis PL, Karaca E, Melquiond ASJ, van Dijk M, de Vries SJ, Bonvin AMJJ. The HADDOCK2.2 web server: user-friendly integrative modeling of biomolecular complexes. *J Mol Biol* (2016) **428**:720–725. doi:10.1016/j.jmb.2015.09.014

13. Livak KJ, Schmittgen TD. Analysis of relative gene expression data using real-time quantitative PCR and the 2(-Delta Delta C(T)) Method. *Methods* (2001) **25**:402–408. doi:10.1006/meth.2001.1262

14. Lieskovská J, Páleníková J, Širmarová J, Elsterová J, Kotsyfakis M, Campos Chagas A, Calvo E, Růžek D, Kopecký J. Tick salivary cystatin sialostatin L2 suppresses IFN responses in mouse dendritic cells. *Parasite Immunol* (2015) **37**:70–78. doi:10.1111/pim.12162

15. Levashina EA, Moita LF, Blandin S, Vriend G, Lagueux M, Kafatos FC. Conserved role of a complement-like protein in phagocytosis revealed by dsRNA knockout in cultured cells of the mosquito, *Anopheles gambiae*. *Cell* (2001) **104**:709–718. doi:10.1016/S0092-8674(01)00267-7

16. Hajdusek O, Sojka D, Kopacek P, Buresova V, Franta Z, Sauman I, Winzerling J, Grubhoffer L. Knockdown of proteins involved in iron metabolism limits tick reproduction and development. *Proc Natl Acad Sci U S A* (2009) **106**:1033–1038. doi:10.1073/pnas.0807961106

17. Long GL, Chandra T, Woo SL, Davie EW, Kurachi K. Complete sequence of the cDNA for human alpha 1-antitrypsin and the gene for the S variant. *Biochemistry* (1984) **23**:4828–4837. doi:10.1021/bi00316a003

18. Mulenga A, Khumthong R, Blandon MA. Molecular and expression analysis of a family of the *Amblyomma americanum* tick Lospins. *J Exp Biol* (2007) **210**:3188–3198. doi:10.1242/jeb.006494

19. Porter L, Radulovic Z, Kim T, Braz GRC, Da Silva Vaz I, Mulenga A. Bioinformatic analyses of male and female *Amblyomma americanum* tick expressed serine protease inhibitors (serpins). *Ticks Tick Borne Dis* (2015) **6**:16–30. doi:10.1016/j.ttbdis.2014.08.002

20. Kim TK, Tirloni L, Berger M, Diedrich JK, Yates JR, Termignoni C, da Silva Vaz I, Mulenga A. *Amblyomma americanum* serpin 41 (AAS41) inhibits inflammation by targeting chymase and chymotrypsin. *Int J Biol Macromol* (2020) **156**:1007–1021. doi:10.1016/j.ijbiomac.2020.04.088

21. Sugino M, Imamura S, Mulenga A, Nakajima M, Tsuda A, Ohashi K, Onuma M. A serine proteinase inhibitor (serpin) from ixodid tick *Haemaphysalis longicornis*; cloning and preliminary assessment of its suitability as a candidate for a tick vaccine. *Vaccine* (2003) **21**:2844–2851. doi:10.1016/S0264-410X(03)00167-1

22. Imamura S, Da Silva Vaz I, Sugino M, Ohashi K, Onuma M. A serine protease inhibitor (serpin) from *Haemaphysalis longicornis* as an anti-tick vaccine. *Vaccine* (2005) **23**:1301–1311. doi:10.1016/j.vaccine.2004.08.041

23. Wang F, Song Z, Chen J, Wu Q, Zhou X, Ni X, Dai J. The immunosuppressive functions of two novel tick serpins, HlSerpin‐a and HlSerpin‐b, from *Haemaphysalis longicornis*. *Immunology* (2020) **159**:109–120. doi:10.1111/imm.13130

24. Toyomane K, Konnai S, Niwa A, Githaka N, Isezaki M, Yamada S, Ito T, Takano A, Ando S, Kawabata H, et al. Identification and the preliminary *in vitro* characterization of IRIS homologue from salivary glands of *Ixodes persulcatus* Schulze. *Ticks Tick Borne Dis* (2016) **7**:119–125. doi:10.1016/j.ttbdis.2015.09.006

25. Leboulle G, Crippa M, Decrem Y, Mejri N, Brossard M, Bollen A, Godfroid E. Characterization of a novel salivary immunosuppressive protein from *Ixodes ricinus* ticks. *J Biol Chem* (2002) **277**:10083–10089. doi:10.1074/jbc.M111391200

26. Ibelli AMG, Kim TK, Hill CC, Lewis LA, Bakshi M, Miller S, Porter L, Mulenga A. A blood meal-induced *Ixodes scapularis* tick saliva serpin inhibits trypsin and thrombin, and interferes with platelet aggregation and blood clotting. *Int J Parasitol* (2014) **44**:369–379. doi:10.1016/j.ijpara.2014.01.010

27. Mulenga A, Tsuda A, Onuma M, Sugimoto C. Four serine proteinase inhibitors (serpin) from the brown ear tick, *Rhiphicephalus appendiculatus*; cDNA cloning and preliminary characterization. *Insect Biochem Mol Biol* (2003) **33**:267–276. doi:10.1016/s0965-1748(02)00240-0

28. Yu Y, Cao J, Zhou Y, Zhang H, Zhou J. Isolation and characterization of two novel serpins from the tick *Rhipicephalus haemaphysaloides*. *Ticks Tick Borne Dis* (2013) **4**:297–303. doi:10.1016/j.ttbdis.2013.02.001

29. Xu Z, Yan Y, Zhang H, Cao J, Zhou Y, Xu Q, Zhou J. A serpin from the tick *Rhipicephalus haemaphysaloides*: involvement in vitellogenesis. *Vet Parasitol* (2020) **279**:109064. doi:10.1016/j.vetpar.2020.109064

30. Tirloni L, Seixas A, Mulenga A, da Silva Vaz I, Termignoni C. A family of serine protease inhibitors (serpins) in the cattle tick *Rhipicephalus (Boophilus) microplus*. *Exp Parasitol* (2014) **137**:25–34. doi:10.1016/j.exppara.2013.12.001

31. Kaewhom P, Sirinarumitr T, Chantakru S, Jittapalapong S. Cloning and characterization of cDNA encoding a serine protease inhibitor from salivary glands of Thai cattle tick (*Boophilus microplus*). *Kasetsart J (Nat Sci)* (2007) **41**:74–80.
